# Aging-Related Mechanisms Underlying Carcinogenesis: Therapeutic Opportunities

**DOI:** 10.14336/AD.2025.0861

**Published:** 2026-09-27

**Authors:** Ana Guijarro, Giulia Allavena, Livia Pisciotta, Alessio Nencioni, Irene Caffa

**Affiliations:** ^1^Department of Internal Medicine and Medical Specialties, University of Genoa, 16132 Genoa, Italy.; ^2^Institute for Research on Cancer and Aging of Nice (IRCAN), CNRS UMR7284, INSERM U1081, Université Côte d'Azur, 06107 Nice, France.; ^3^IRCCS Ospedale Policlinico San Martino, 16132 Genoa, Italy.

**Keywords:** Aging, aging-delaying therapies, clinical trials, inflammation, senescence, somatic mutations, tumorigenesis

## Abstract

Aging is a gradual loss of tissue homeostasis that leads to impaired physiological organ functions and constitutes a major risk factor for cancer initiation and progression. Despite advances in anti-tumor therapies, cancer remains the second leading cause of death worldwide. The rising incidence of cancer is intimately associated with increased lifespan and the growing proportion of older adults, with 64% of cancers diagnosed in people aged 60 and above. Mechanisms underlying aging include accumulation of somatic mutations, deficient DNA damage repair machinery, telomere shortening, enhanced genomic instability, epigenetic alterations, loss of heterochromatin, chronic low-grade inflammation, mitochondrial dysfunction, cellular senescence and its associated secretory phenotype, stem cell exhaustion, aberrant intercellular communications, remodeling of extracellular matrix and microenvironment, impaired nutrient sensing and alterations in the proteome. Additionally, dysregulation of the circadian clock, the endocannabinoid system and the microbiota may also play important roles. Given that many of these processes are also crucial for cancer development, it is widely admitted that aging and cancer are tightly interconnected. Consequently, many therapies aimed at delaying or mitigating aging, such as physical exercise, specific dietary regimens, chronotherapy, epigenetic drugs and senotherapeutics, might also prevent or retard cancer development and progression and reduce the side effects of cancer therapies. However, special caution must be taken in older cancer patients due to their comorbidities and possible frailty, selecting specific patients’ treatments and balancing the extended survival with the preservation of independence and quality of life.

## 1. Introduction

Aging can be defined as a progressive series of physiological alterations that lead to the loss of tissue homeostasis and subsequent decline of biological functions, ultimately predisposing to multiple pathologies, including emphysema, osteoarthritis, cardiovascular diseases, cognitive decline, dementias and malignancies, collectively referred to as age-related diseases (ARDs) [1]. Traditionally, the accumulation of nuclear and mitochondrial DNA damage, genomic instability, loss of heterochromatin [1] and somatic mutations [2] has been considered as a primary cause of aging [3, 4]. However, this view has evolved in light of evidence showing that certain features of aging can be reversed by anti-aging interventions and that lifespan can be extended [5-10].

Chronological age, defined as the time elapsed since birth, does not always correspond to biological age, which reflects the physiological state of tissues and organs as they accumulate molecular and cellular damage. Biological age is influenced by a variety of different biological and physiological factors, including lifestyle, nutrition and genetics [11]. The discrepancy between biological and chronological age is defined as “age acceleration”, and subjects displaying a positive age acceleration are more prone to earlier onset of functional decline [12, 13]. This distinction between chronological and biological age is particularly relevant in oncology, where therapeutic decisions for elderly cancer patients should be based on both chronological and biological age [14, 15]. Furthermore, it is also important to distinguish healthy aging [14, 16] from unhealthy aging [17], which is characterized by premature senescence, senescence-associated secretory phenotype (SASP), chronic low-grade inflammation (“inflammaging”) and ARDs.

Cancers comprise a heterogeneous group of diseases in which normal cells acquire specific somatic “driver” mutations in tumor-suppressor genes and/or proto-oncogenes, leading to their transformation into malignant cells capable of uncontrolled proliferation, tumor formation, and metastatic spread. Despite notable therapeutic progress, cancer remains the second leading cause of death worldwide, after cardiovascular diseases [18], and the fourth most common cause of death among the very old [19]. In 2020, 64% of newly diagnosed cancer cases occurred in people aged 60 and above, while those aged 85 years or older accounted for more than 6% of all cancer cases [18, 20], highlighting aging as a main, well-recognized, risk factor for cancer occurrence [18, 21]. According to the World Health Organization, the global population aged 60 years and older will reach 2.1 billion by 2050, with the number of subjects aged 80 years or above expected to triple to 426 million [22]. Therefore, given the ongoing increase in life expectancy [23-26], global cancer incidence is expected to rise to 20.7 million new cases and 12.7 million deaths by 2040 [18, 27], accounting for a major burden on healthcare systems. Furthermore, aging influences both cancer incidence [28-31] and prognosis [32-36], with older patients generally experiencing shorter progression-free survival (PFS) and lower overall survival (OS), underscoring the biological interconnection between aging and tumorigenesis.

At cellular level, aging is driven by mechanisms such as impaired DNA damage repair, telomere shortening, enhanced genomic instability [37], cellular senescence [38], reduced basal autophagy [39], epigenetic alterations, mitochondrial dysfunction, stem cell decline, aberrant intercellular communications, changes in the extracellular matrix (ECM) and microenvironment composition, impaired nutrient sensing and alterations in proteome composition [1, 40, 41]. Remarkably, many of these mechanisms also underpin cancer development [42-45], despite the apparent contrast between the “loss of function and fitness” seen in aging cells [46] and the “gain of function and fitness” observed in cancer cells [1]. As highlighted by Trastus *et al.* in their recent comprehensive review, the relative contribution of different aging mechanisms and pathways in tumorigenesis is complex and context-dependent [47].

## 2. Biological systems and mechanisms altered by aging with potential impact on carcinogenesis

### 2.1. Somatic mutations and genome instability

#### 2.1.1. Progressive acquisition of somatic mutations

Somatic mutations arise due to error-prone DNA repair, replicating damaged DNA, stochastic errors by DNA polymerases or telomere shortening [48-52]. Additional mechanisms include the accumulation of oxidative DNA damage due to mitochondrial dysfunction [53], defective cell cycle checkpoints [54] and exogenous environmental factors, such as radiation and chemicals [55]. While such mutations are the driving force of genetic diversity and evolution, they also represent the key form of genome instability, a hallmark of both aging [1, 56] and diseases, particularly cancer [46, 57] (Fig. 1A).

In somatic cells, genome instability typically alters regulatory sequences, impacting cell proteome and homeostasis, which eventually leads to functional decline in tissues and organs. However, mutations can also affect protein-coding genes [58] or key regulatory elements, enabling cells to acquire oncogenic properties such as growth factor independence and enhanced metastatic potential [46]. According to the somatic mutation theory of aging, the cumulative burden of such mutations results in cell senescence, cell death or loss of function, thereby promoting aging [59, 60]. When these mutations occur in stem cells, they can lead to their depletion or dysfunction, contributing to both aging and malignant transformation [56, 58] (Fig. 1C).

Somatic mutations are broadly acknowledged as drivers of tumorigenesis [45, 61], with some “neutral/passenger mutations”, which do not directly contribute to the tumor phenotype, and others “driver mutations”, which confer a selective growth advantage that promotes clonal expansion [55, 62-66]. Somatic mutations can occur in cell cycle genes, resulting in the activation of oncogenes (e.g., *ERBB2*, *EGFR*, *MCL1*, *MYC, KRAS, BRAF, CCNC, CCND1*) [67-74], or the inactivation of tumor-suppressor genes (e.g., *RB1*, *PTEN*, *CDKN2A/B, p16*) [62, 64] (Fig. 1A). To add another layer of complexity, a third class of mutations known as “latent drivers” [75] may become drivers at a specific stage of cancer progression or when combined with other mutations [66]. The mutational heterogeneity within tumors further enables them to evade immune system surveillance and resist therapeutic intervention.

Notably, cancer-associated mutations accumulate with age, and in elderly adults, the prevalence of cancer-associated mutations in normal tissue can approach 100% [45]. For instance, clonal patches of somatic mutations in normal skin often involve well-known skin cancer drivers, such as *NOTCH* family, *TP53*, *FAT1* and *RBM10* [76]. Likewise, many mutations found in normal cells are additionally found in cancers from the same tissues. For example, in the esophagus, up to 80% of cells in older individuals harbor *NOTCH1* mutations, also common in esophageal cancer [77]. A systematic study of somatic mutations, carried out in thousands of cancer patients, demonstrated that the mutations frequency increases exponentially with age [78], with distinct mutation spectra associated with aging [79]. These findings were supported by studies on adult stem cells of the small intestine, colon and liver, which exhibited age-related mutation burdens reflective of tissue-specific cancer mutation profiles [80]. Similar overlap in the somatic mutation pattern of normal aging tissue and its associated cancer has been reported by Hoang *et al*., reinforcing the view that age-related mutational processes are central to both aging and oncogenesis [81].

**Figure 1.**
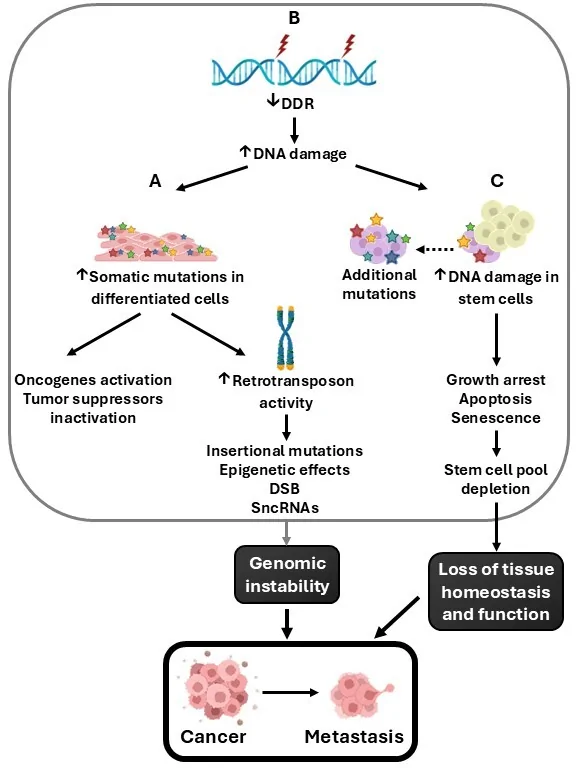
**Processes that occur during aging that promote cancer initiation and progression: somatic mutations**. (**A**) Somatic mutations can result in the silencing of tumor-suppressors or the activation of oncogenes driving tumorigenesis. The increased activation of retrotransposons leads to insertional mutations, genomic instability, epigenetic alterations, double strand breaks (DSB) and expression of small noncoding RNAs (SncRNAs), promoting carcinogenesis. (**B**) Aging is associated with a defective DNA damage repair response (DDR), which results in accumulation of DNA damage. C. With aging, stem cells accumulate DNA damage and mutations that lead to growth arrest, apoptosis or senescence, resulting in a reduction of the stem cells pool. Some of these damaged cells can evade control pathways by acquiring additional mutations, they continue to proliferate and provide a favorable environment for the development of new malignancies.

Despite this, determining the causative effect of aging-related mutations on cancer initiation remains challenging, since mutations can also arise after neoplastic transformation and during tumor progression. As mentioned above, driver mutations may result from random errors made during normal DNA replication, and this has been proposed as a major determinant of tissue-specific cancer risk [82]. Approximately three mutations are estimated to occur during each human stem cell division [83, 84]. Tomasetti *et al.* reported that the number of these random mutations increases with age, and that lifetime cancer risks among 25 different tissues strongly correlate with the total number of stem cell divisions in those tissues [82]. Mouse studies corroborate this hypothesis, showing that both stem cell proliferation and DNA damage increase cancer risk [85].

Thus, cancer development likely results from a synergistic interaction between intrinsic factors (e.g., stem cell divisions, replication errors), genetic predisposition and extrinsic factors (e.g., environmental mutagens) [85]. The relative contribution of these three factors to cancer development is difficult to determine. After accounting for environmental and hereditary influences, a comprehensive genomic study attributed two-thirds of the mutations in human cancers to replication-associated errors [65]. Despite this, many cancers can still be preventable, as tumor formation generally requires more than one mutation [65, 86], and others are still amenable to secondary prevention through early detection and intervention [65].

Furthermore, tumor sequencing studies revealed hundreds to thousands of shared somatic mutations in tumor cells, with mutation burden correlating with patient age [78, 80, 87-89]. Remarkably, over half of these mutations appear to precede tumor initiation, emphasizing the impact of age-related mutation accumulation [84]. Across all tested cells and tissues, aging leads to the accrual of single-base substitutions (SBSs), single-nucleotide variants (SNVs) and small insertions/deletions (indels or IDs) [90]. The rate and spectrum of these mutations vary by cell types, genomic region and tissue [91-95], resulting in genomic mosaicism, where tissues are composed of cells with slightly different genomes [96], a dynamic phenomenon in both aging and disease [45]. It has been proposed that accumulated mutations act as a “genomic burden” that gradually impairs cellular functions [97]. High mutational load can trigger cell cycle arrest, senescence, or apoptosis, serving as a defense mechanism against malignant transformation [98]. However, in some contexts, these mutations contribute to tumor heterogeneity, treatment resistance, and poor prognosis. Furthermore, a role of copy number variations (CNVs) in tumor onset, progression, heterogeneity, treatment response and prognosis has been proposed [99-102].

#### 2.1.2. Chromosomal rearrangements: Structural variations and chromothripsis

Historically, the extensive genomic alterations observed in tumors have been attributed to a stepwise process in which driver mutations progressively accumulate over time [64]. Nevertheless, the work by Stephens *et al.* demonstrated that certain chromosomal rearrangements in cancer genomes could not be fully explained by this “progressive” model. Instead, they might be the result of a single “catastrophic event” occurring within a single cell cycle, leading to multiple chromosomal breaks followed by random reassembly [103]. The emergence of such catastrophic mechanisms is closely linked to genomic instability [104].

A particularly important class of somatic mutations in carcinogenesis is structural variations (SVs), defined as genomic alterations (e.g., deletion, amplification or rearrangements of genomic segments) ranging from a few base pairs to kilobase and megabase-sized fragments [103, 105-112]. Somatic SVs, which are present only in a subset of cells, arise *de novo* in individual somatic cells and accumulate over successive cell divisions across the lifespan [111, 113]. Importantly, SVs frequently mediate gain- or loss-of-function of cancer-related genes and therapeutic targets [114].

Recent studies analyzing SVs patterns and signatures across the Pan-Cancer Analysis of Whole Genomes (PCAWG, 2,559 whole cancer genomes across 38 tumor types) revealed that 2,429 tumors harbored at least one somatic SV [109]. These studies highlighted marked variability in the SVs overall frequency and distribution across both tumor types and patients within a given tumor type [110, 115-118].

Another single “catastrophic event” is chromothripsis [103], in which one or more chromosomes shatter into multiple fragments and are then massively rearranged in a single event. Chromothripsis is a major driver of cancer initiation [119, 120] contributing to oncogenes overexpression [103, 120-122], loss of tumor-suppressor functions [103, 116, 123] and formation of fusion genes [123-125], and is also involved in the development of resistance to several anti-tumor drugs, including immunotherapy, platinum genotoxins and anti-angiogenesis drugs [126, 127]. Moreover, the transcriptional profile of tumors displaying chromothripsis has been associated with an immune evasion phenotype [127].

Whole genome sequencing has documented chromothripsis frequency exceeding 50% in certain tumors [128] and an average prevalence higher than 30% across cancer types [129], though the occurrence varies widely depending on tumor types [120]. The association between age and chromothripsis seems also to be tumor-specific: in acute myeloid leukemia (AML) [130, 131] and in a large multi-tumor screen [131], incidence increases with advanced age, whereas no correlation with age has been observed in prostate cancer (PC) [132]. In osteosarcomas, chromothripsis was detected throughout all ages, with incidence being highest at adolescence and old age [133].

#### 2.1.3. Mini- and microsatellite copy number alterations

A distinct category of SVs comprises mini- and microsatellite copy number alterations. Given the positive correlation between lifespan and DNA repair capacity [134], it has been proposed that age-related deficiency in the cellular mechanisms of DNA repair [i.e., nucleotide excision repair pathway (NER), nonhomologous end-joining pathway and mismatch repair (MMR) system] [42, 135-138] could contribute to the progressive accumulation of genetic damage [135] (Fig. 1B).

In particular, impairment of the MMR system with age [139] is evidenced by the acquisition of microsatellite instability (MSI) [138], which may predispose individuals to cancer. Indeed, excessive MSI has been strongly linked to colorectal cancer (CRC), often due to loss of the DNA MMR system [140]. The biology of MSI-high (MSI-H) in elderly CRC patients is attributed to hypermethylation of the MMR gene promoters (e.g., MLH1). Age-related MLH1 promoter methylation in normal colonic mucosa is closely linked to age and to the development of sporadic CRCs [141].

Approximately 15% of spontaneous CRCs display MSI, being the vast majority of these caused by epigenetic silencing of MLH1 [142]. A similar age-related increase in MSI and the microsatellite variability have been documented in gastric lymphoma [143]. Microsatellite mutations in human blood also increase with age [137, 138], with the highest rates seen in tumors with MMR deficiency, such as endometrial, rectal and stomach cancer, but microsatellite mutations were also detected in cervical and breast tumors [144].

Defects in DNA MMR machinery correlated with specific driver mutations in *PIK3CA* and *FBXW7* in CRC and stomach cancer, and with mutations in *KRAS* in uterine carcinoma and stomach cancer [145]. Moreover, deficiency in the DNA-proofreading system has also been reported in uterine carcinoma and CRC and was associated with mutations in *PTEN* and in *APC* genes, indicating that *POLE* defects may cause characteristic driver lesions in these cancer types [87].

Given that (i) there is an association between cancer and MSI, (ii) most malignancies occur in old people, and (iii) DNA repair capacity decreases with age, it has been postulated that defects in MMR mechanisms may contribute to the high cancer incidence in the elderly and may affect the efficiency of cellular functions.

#### 2.1.4. Transposable elements

Transposable elements (transposons, TEs), particularly retrotransposons that mobilize via an RNA intermediate [146], are mobile genetic elements capable of copying and inserting themselves into new genomic locations. This "copy-and-paste" mechanism, which leads to the generation of repetitive sequences, not only contributes to intra- and inter-individual variation, but also acts as a potent insertional mutagen [147]. Beyond this mutagen role, retrotransposons can also impact gene regulation, epigenetic landscapes, genomic stability, and the expression of small non-coding RNAs (sncRNAs) [147] (Fig. 1A), conditions also involved in cancer development and progression [148].

Among retrotransposons, long interspersed nuclear element (LINE-1, L1) is particularly significant as it is the only protein-coding mobile genetic element in the human genome [149]. L1 expression drives chromosomal rearrangements, copy-number alterations and subclonal copy-number heterogeneity, contributing to chromosomal instability [150]. L1 activation has been detected in approximately 50% of human tumors [151, 152] and is considered a potent mutagenic force [153]. Retrotranspositions of L1 has significantly higher frequencies in certain cancers: 97% of invasive breast carcinomas, 93.5% of high-grade ovarian carcinomas, 89% of pancreatic ductal adenocarcinomas and 61% of carcinomas originating in the endometrium, biliary tract, esophagus, bladder, head and neck, lung and colon. Conversely, lower levels are seen in kidney, liver, cervix and prostate tumors (~24%) [154]. Particularly, high L1 retrotransposition rates are found in tumors with *TP53* mutations, underscoring a link between loss of tumor-suppressor function and increased transpositional activity [152, 154, 155].

Somatic L1 retrotranspositions can impact carcinogenesis by promoting amplification of oncogenes, such as *CCND1* [152], activating proto-oncogenes like *HDAC9* and *RUNX1T1*, or by disrupting tumor-suppressors as *CYLD, APC, MCC, PTPRM, PCM1, CDH11* and *ROBO2* [156-160].

A noteworthy finding is the positive correlation between patient age and the number of L1 insertions in sporadic colon cancers, suggesting that aging and genomic instability may have cumulative effects on retrotransposition. Hypomethylation, commonly observed in aged and tumor tissues, may create a permissive environment for L1 activation [159].

Furthermore, a big body of evidence indicates that retrotransposons are not only a marker of aging but also active contributors to the aging process and ARDs [161-163], since they are reactivated in cell senescence and with age [163, 164]. The deleterious effects of retrotransposons linked to aging are primarily due to: (i) DNA damage and associated genome instability, (ii) genetic and epigenetic effects and (iii) triggering of immune pathways associated with detection of retrotransposon nucleic acids [161].

The expression and activity of L1 are tightly regulated by various cellular processes, including repressive epigenetic modifiers [165], DNA repair [166], innate immune response [167] and autophagy [168]. Aging disrupts these pathways, leading to L1 de-repression [169-171], often in a cell-type-specific manner [172, 173]. Additionally, aging promotes an environment that enhances L1 expression, particularly through oxidative stress [174, 175] and downregulation of TE repressive factors [176]. These changes are accompanied by epigenomic remodeling and global DNA hypomethylation [1, 177-179], closely resembling patterns found in cancer genomes [180].

Senescent cells, which accumulate with age [181], further increase L1 activity [182]. De Cecco *et al.* showed that in senescent mouse and human cells, L1 elements become transcriptionally de-repressed and trigger a type-I interferon (IFN-I) response that contributes significantly to the SASP. This response is antagonized by nucleoside reverse transcriptase inhibitors (NRTIs), suggesting a link between L1 activity and inflammaging [183]. Importantly, enhancing heterochromatin formation or silencing retrotransposons can reduce age-related L1 activation and even extend lifespan [184].

However, senescence is not the only contributor to the increased retrotransposon activity observed in aging, given that a robust retrotransposon activation with aging is observed in several species devoid of cell senescence [182, 185] and in mouse tissues with a low burden of senescent cells [183, 186]. Collectively, these studies indicate that retrotransposons causally contribute to the aging process and highlight them as potential targets to promote healthy longevity.

#### 2.1.5. Mutational signatures

It has been estimated that human tissues generally accumulate approximately 40 mutations/genome/year, regardless of tissue type [80, 87]. Three landmark studies by Alexandrov *et al.* identified distinct “mutational signatures”, each representing the footprint of a distinct mutagenic process that generates distinct combinations of mutation types, and provided further support for an underlying mutational process that gives rise to age-associated mutations [79, 187, 188]. The authors initially identified 21 mutational signatures operating over 30 cancer types [79], later, using a redefining approach, they identified 33 distinct mutational signatures, of which 29 were validated [87]. Among these signatures, signatures 1 and 5 displayed a correlation between mutation burden and age at diagnosis across many different cancer types [79, 87]. For both signatures, the mutation rate showed substantial variation among different cancer types and, in the case of signature 1, it seems to be determined by the cell proliferation rate [87]. Importantly, the mutation rates of both signatures do not correlate, indicating that they could have different origins.

A posterior study, which comprises 2,780 PCAWG whole genomes, also revealed a positive correlation between age and the number of mutations for several signatures [109]. Similarly, Temko *et al.* [145] showed that 6 associations between mutational processes and driver mutations involved signatures that are known to correlate with age at diagnosis [87], highlighting the important role of aging-related processes in cancer initiation.

### 2.2. DNA damage and DNA damage repair

The correction of DNA alterations induced by exogenous and endogenous environmental insults involves several complex DNA repair mechanisms, which trigger cell cycle checkpoints and allow DNA damage repair, hence reducing the probability of tumor development [189, 190]. Upon severe DNA damage, temporary/permanent cell cycle arrest or apoptosis may occur. While protecting against cancer, these responses can also have adverse long-term effects, such as the depletion of stem-cell reservoirs, contributing to aging (Fig. 1B).

Extensive evidence links DNA damage accumulation, defective DNA repair machinery and aging [3, 42, 134-136, 166, 191], and has pointed out the age-dependent accumulation of DNA damage as a contributor to the impairment of cellular and organ functions and the higher rate of diseases, such as cancer, in the elderly (Fig. 1B). Similarly, aberrations in DNA damage signaling and repair are closely associated with cancer [190, 192, 193]. Defective DNA damage repair pathways may arise from genetic inactivation (e.g., mutations in MMR genes or homologous recombination repair genes) or from epigenetics (e.g., silencing by promoter hyper-methylation, histone post-translational modifications) [190]. Genes encoding components of the DNA damage response (DDR) are among the most frequently mutated genes in cancer [194, 195] and their inactivation promotes a “mutator phenotype”, leading to the accumulation of additional cancer-driving genomic aberrations [196-198]. Experimental work carried out in a zebrafish *MYCN* transgenic model shows that mutations in the DDR genes, including *Brca2*, *Atm* and *Palb2*, enhance neuroblastoma formation and metastasis and upregulates cell cycle checkpoint and DNA damage repair signatures [199].

In addition, DNA repair deficiencies have been proposed to accelerate the onset of a wide variety of ARDs [200]. Accordingly, mutations in DNA repair pathways (e.g., *POLH*, XPA/B/C/G, DDB2, *WRN, BLM, BRCA2, FANCA*, *ATM, RECQL4, ERCC4/6*) can also result in several premature aging (progeroid) syndromes (which mimic many of the characteristics of human aging), including Werner syndrome (WS), Bloom syndrome (BS), Rothmund-Thomson syndrome, Cockayne syndrome, xeroderma pigmentosum (XP), trichothiodystrophy, Fanconi anaemia (FA), Seckel syndrome, ataxia telangiectasia (AT), ataxia telangiectasia-like disorder, cerebroretinal microangiopathy with calcifications and cysts and Nijmegen breakage syndrome [201]. Many of these syndromes not only display accelerated aging phenotypes, but also predispose to cancer, as seen in XP [202], WS [203], BS [204], FA [205], AT [206]. It has been suggested that the presence of DNA lesions can induce significant rearrangements of intracellular signaling pathways, metabolism and autophagy [207], changes that are observed in physiological aging, underscoring the central role of genome maintenance in cellular responses and, ultimately, aging.

Stem cells, with their long lifespan, are particularly vulnerable to replication-induced mutations and, although equipped with mechanisms to safeguard genomic integrity, they accumulate DNA damage with age [208]. Aged mesenchymal stem cells display enhanced DNA damage and a downregulation of genes associated with base excision repair (BER) and NER, MMR, and double-strand break repair (DSBR). In addition, hematopoietic stem and progenitor cells show an age-related decline in DSBR efficiency, fostering genomic instability and mutagenic burden, which can lead to bone marrow malignancies [41].

Similar to other tissue-specific stem cells, hematopoietic stem cells (HSCs) are susceptible to aging-related stress, losing self-renewal and regenerative capacities [209, 210], leading to a reduced blood cell production and immune dysfunction [211]. Aging also fosters clonal hematopoiesis, whereby genetically mutant HSCs expand selectively [209-211], conferring a modest but significant risk of leukemia [45]. DNA damage is a primary driver of HSC aging [212, 213], with aged HSCs exhibiting a 2- to 3-fold increase in accumulated DNA damage [213, 214]. In addition, mutations in epigenetic modifiers are often observed in healthy elderly individuals, altering the epigenetic landscape of HSCs and influencing both aging and hematological cancer susceptibility [212, 215]. Of note, changes in epigenetic methylation landscapes are a hallmark of hematological cancers and promote their initiation and progression.

Damaged stem cells undergo growth arrest, apoptosis or senescence leading to decreased overall number and/or functionality of the stem cells, with the subsequent reduction of regenerative capacity and loss of tissue homeostasis, contributing to cancer development (Fig. 1C). Furthermore, some of these damaged cells can evade control pathways acquiring additional mutations (e.g., reactivation of telomerase, loss of *p16^INK4a^*) enabling unchecked proliferation and creating a permissive environment for new malignancies (Fig. 1C) [208].

Accretion of DNA damage contributes to aging not only by impairing tissue maintenance but also by promoting inflammation [191], a hallmark of aging and a driver of many ARDs, which will be discussed later. Notably, many progeroid syndromes exhibit chronic inflammation states [201]. Age-related DNA damage accumulation can activate the inflammation pathway via the cGAS-STING axis or NF-κB activation by ATM, two mechanisms also triggered by cellular senescence, transposon activation and the accumulation of persistent R-loops [191]. In turn, chronic inflammation perpetuates itself through cytokine secretion, which promotes redox stress, activates DDR, and leads to senescence and SASP production, thus establishing a vicious cycle of DNA damage and DDR-mediated senescence and inflammation, accompanied by the constitutive activation of the immune system [216].

### 2.3. Telomere attrition

Telomere shortening is a well-established feature of normal human aging, providing a barrier to tumor growth and contributing to cell senescence [217]. Indeed, age-dependent telomere attrition has been reported in 21 out of 24 human tissues examined [218]. Furthermore, two progeroid syndromes, Hoyeraal-Hreidarsson syndrome and dyskeratosis congenita (DKC), are linked to mutations in components of the telomerase complex (i.e., *ACD* [219] and *RTEL1* [220], respectively), that cause accelerated telomere attrition [216-219]. Notably, DKC, which involves mutations also in other telomerase-associated genes, including *WRAP53*, *TERC*, *CTC1* and *TERT*, exhibits increased cancer predisposition [221].

In non-proliferating, post-mitotic tissues, telomere dysfunction may arise from DNA damage within telomeres. Such lesions, which appear to be irreparable, lead to persistent DDR signaling [222]. This unrelenting telomeric DDR (tDDR) activation enforces a chronic senescent phenotype [223]. The tDDR activation is also triggered by age-related telomere shortening, and therefore is often causally linked to many aging hallmarks, including aberrant nutrient sensing, proteostasis loss, mitochondrial dysfunction, defective autophagy and epigenetic deregulation (Fig. 2A). This has led to the suggestion of a “telomere-centric” mechanistic rationale for explaining diverse aging-associated processes [218].

Evidence supporting tDDR as a driver of aging and ARDs comes from observations that conditions known to accelerate aging, including mitochondrial dysfunction, impaired autophagy, chronic inflammation and obesity, promote tDDR. Conversely, interventions known to extend healthspan, including exercise, dietary restriction, 17β-estradiol and rapamycin administration, have been shown to blunt tDDR [223]. It has been proposed that it is not telomere deregulation *per se* that drives aging and ARDs, but rather is tDDR, activated by telomere dysfunction, that causes cellular senescence, which, through SASP induction, contributes to the age-related loss of tissue functions [224] (Fig. 2A).

**Figure 2.**
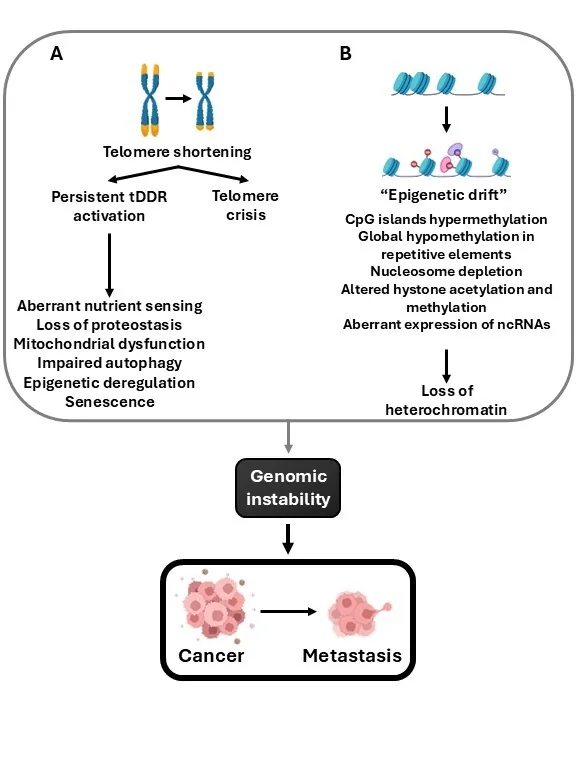
**Processes that occur during aging that promote cancer initiation and progression: telomere shortening and “epigenetic drift”**. (**A**) Telomere shortening induces persistent activation of the telomeric DNA damage response (tDDR) that is causally linked to many aging hallmarks that promote cancer development. In addition, loss of telomere protection of the ends of chromosomes can result in telomere crisis, which is a state of extensive genome instability that can favor cancer initiation and progression. (**B**) Aging phenotype is associated with epigenetic drift, that can result in a higher risk of cancer development.

Telomere shortening exerts a paradoxical effect on cancer development. On one hand, critically short telomeres can suppress tumorigenesis by activating ATR and ATM kinases at unprotected chromosome ends, leading to senescence or apoptosis. On the other hand, loss of telomere protection can result in telomere crisis [225], a state of extensive genome instability that facilitates cancer progression by inducing chromothripsis, kataegis and tetraploidization [226] (Fig. 2A). Escape from telomere crisis typically requires telomerase activation, which restores telomere integrity and replicative potential. This results in a telomerase-positive, transformed cell with a massively rearranged, but stabilized, genome enriched with new and potentially tumorigenic genetic alterations [227].

Using a zebrafish model, Carneiro *et al.* demonstrated that, as age progresses, telomeres shorten to critical lengths only in specific tissues (gut and muscle), independently of their proliferative rates. This telomere-associated DNA damage not only impairs local tissue function, but also facilitates the onset of cancer, cachexia and infection in other tissues. The onset of such conditions correlated with the age at which most tissues started to exhibit short telomeres, and telomerase mutants (tert^-/-^) zebrafish recapitulated these diseases prematurely [228].

Similarly, Lopes-Bastos *et al.* investigated melanoma progression and found that tumors display similar incidence and invasiveness in tert^-/-^ and telomerase wild-type zebrafish. However, at later stages, tert^-/-^ tumors show reduced cell proliferation, enhanced apoptosis, melanocyte differentiation and immune cell infiltration. These results suggest that telomerase deficiency constrains melanoma progression through both tumor-autonomous and non-tumor-autonomous mechanisms [229].

### 2.4. Epigenetic changes

Aging profoundly remodels the epigenome, defined as the ensemble of heritable DNA and histone modifications that regulate chromatin spatial organization, modulating gene expression. The cumulative modifications occurring during aging are termed “epigenetic drift” [76] (Fig. 2B). The foremost age-associated epigenetic modifications encompass CpG islands hypermethylation at specific loci (e.g., Polycomb gene) alongside global hypomethylation in repetitive elements [172]. In addition, a general nucleosome depletion, altered histone acetylation and methylation, telomere shortening and an aberrant expression of ncRNAs are observed with aging [230, 231]. Collectively, these modifications reduce heterochromatin integrity and increase genomic instability (Fig. 2B). Notably, epigenetic age, as measured by CpG methylation profiles, has been proven distinct from cellular senescence, telomere attrition and genomic instability, yet it correlated with mitochondrial activity, nutrient sensing and stem cell composition [232].

Epigenetic reprogramming is also recognized to be one of the earliest and most comprehensive genomic aberrations happening during carcinogenesis [233], being associated with a broad spectrum of cancers [234]. Hypermethylation of the promoters of tumor-suppressor genes (e.g., *Rb*, *RUNX3*, *p14*, *p15*, *p16*, *p21*, *TIGI*), regulatory genes (e.g., RAS association domain family 1A), retinoic acid receptor β, and DNA repair system genes (e.g., *BRCA1*, *MGMT*) can result in their inactivation, genetic instability and subsequent cancer development [235]. Conversely, DNA hypomethylation contributes to carcinogenesis by activating oncogenes, inducing microsatellite instability, reactivating TEs within the genome, and altering therapy responses [236]. Interestingly, aging stem cells, which acquire an abnormal methylation pattern that stabilizes stemness, are similarly predisposed to malignant transformation [237]. Of note, parallel genetic mutations, often observed in leukemia, further support the clonal selection of altered stem cells during aging, thereby linking epigenetic drift to age-associated carcinogenesis [89].

Notably, the epigenetic drift reported in aged tissues mirrors alterations observed in tumorigenesis [238-241], with aging and cancer sharing overlapping epigenetic signatures [242]. For instance, histone acetylation changes are associated to aberrant metabolism in aging animals, potentially impacting global gene expression [243]. Moreover, the age-driven accumulation of epigenetic modifications has been associated with BRAF-driven tumorigenesis [244, 245], while mammary tissue harboring BRCA1/2 mutations, which predispose to BC, showed an accumulation of epigenetic modifications [246]. Specific epigenetic mutations, such as hotspots of CpG methylation in *TP53*, account for ~37% of somatic *TP53* mutations, underscoring that these mutations are strongly involved in cancer development [247]. Similarly, L1 hypomethylation in colorectal adenomas correlates with a higher CRC risk [248].

The strong relationship between epigenetic changes and biological aging has led to the development of the so-called “epigenetic clock”, tens of different algorithms that use methylation levels at selected CpGs to estimate biological age in different tissues and organisms [249-252]. The discrepancies between chronological and epigenetic age is called “epigenetic age acceleration”. A positive epigenetic age acceleration indicates that a tissue is aging faster than expected by the chronological age and it has been hypothesized to be associated with the risk of ARDs, including cancer [250, 253, 254].

Several studies have recently explored the association between the accelerated epigenetic clock and tumorigenesis [246, 253, 255-258]. Among these, the epiTOC (Epigenetic Timer Of Cancer) is particularly notable. Based on the methylation status of Polycomb genes’ promoters, epiTOC predicts cancer risk and is accelerated not only in cancer, but also in preinvasive lesions, in normal epithelial cells at risk of neoplastic transformation and normal epithelial cells exposed to carcinogens [259]. Lung cancer susceptibility has been associated with intrinsic epigenetic age acceleration, with smokers showing a higher oncogenic risk [260]. Moreover, methylomic-drift of CpG island in premalignant esophageal lesions has been used by Curtius *et al.* to predict progression to esophageal adenocarcinoma [261].

However, findings across different epigenetic clocks and cancer types are not always consistent [255, 262]. For example, metagenomic studies reveal that the GrimAge acceleration may be the best indicator of CRC risk, while other clocks show less consistent association [262]. As suggested by Liu *et al.*, such discrepancies likely reflect the fact that distinct clocks capture different aspects of biological aging, influenced by tissue and population variability [255]. Therefore, more accurate clocks are being developed to improve the prediction of cancer associated risks [263-266].

A recent work studied the functionally enriched modules of age-related and cell type-specific DNA methylation sites associated with senescence, proliferation and stem cell fate, and their association with current and future cancer risk. It identified consistent age-related patterns, exacerbated in cancer, that can be modified by carcinogenic exposures and are mitigated by risk-reducing interventions. Intriguingly, within the same individual, cancer may elicit an acceleration of aging in a tissue at risk but a deceleration of aging in other tissues [267].

### 2.5. Microenvironment and senescence

Apart from the gradual accretion of oncogenic mutations, malignant tumorigenesis also necessitates a permissive and supportive tissue environment in which mutant cells can fully express their neoplastic phenotype [268, 269]. Indeed, while genetic alterations in tumor cells are crucial for the initiation of malignancy, a dynamically evolving tumor-associated ECM is critical for tumor progression because it influences virtually every behavioral facet of stromal and tumor cells. These facets include uncontrolled proliferation, evasion of growth suppression, death resistance, replicative immortality, initiation of invasion, avoidance of immune destruction, chronic inflammation, enhanced angiogenesis and deregulation of cellular energetics, all of them well-established hallmarks of cancer [46, 270, 271].

During aging, components of the ECM become damaged through crosslinking, fragmentation, glycation and enhanced protein aggregation. These cumulative changes impair organ function, contributing to the subsequent development and progression of ARDs [272, 273]. Also, neoplastic progression could be promoted by cellular and molecular alterations of components of the tumor microenvironment (TME) induced by stromal aging and senescence. Experimental evidence shows that neoplastic transformed rat liver epithelial cells, which displayed low tumorigenic potential when transplanted into the livers of young adult rats, exhibit increasing tumorigenicity in the livers of older rats [274]. Likewise, placing tumor cells into an embryonic ECM reprograms them to lose their tumorigenicity and metastatic potential [275]. Inversely, placing aged stem cells or senescent cells in a “younger” ECM rejuvenates these old cells [272]. In the same line, a recent study carried out in syngeneic estrogen receptor (ER)+ breast cancer (BC) mouse models shows that dormant disseminated tumor cells (DTCs) maintain a dormant phenotype in young mice but accelerate metastatic outgrowth in an aged or fibrotic microenvironment [276]. Similarly, aged lung microenvironment constitutes a permissive niche for efficient outgrowth of DTCs, while aged skin inhibits melanoma growth but promotes dissemination [277].

WNT5A has been reported as an activator of dormancy in melanoma disseminated cancer cells within the lung [278]. Age-induced reprogramming of lung fibroblasts enhances their secretion of the soluble WNT antagonist sFRP1, which suppresses WNT5A expression in melanoma cells, enabling efficient metastatic outgrowth. Taken together, these findings indicate that progressive changes in the microenvironment, associated with increasing age, allow tumor formation by providing a less suppressive, and in many cases overtly supportive, microenvironment for expression of the tumorigenic phenotype. This supports the widely discussed notion that the high incidence of cancer linked to aging may result from a dual mechanism: both the accretion of somatic mutations in tumor cells [279] and age-related pro-oncogenic changes in the surrounding tissue milieu [280, 281].

The effects of this aged milieu on cancer can be attributed mostly to three interrelated factors: (i) biomechanical changes in the stromal microenvironment, (ii) senescent stromal cells and (iii) alterations in the secretome and the immune microenvironment [282, 283]. The TME that surrounds cells in solid tumors consists of ECM, composed primarily of structural proteins such as elastin, laminin, collagen and fibronectin, as well as diverse cell types (e.g., endothelial cells, smooth muscle cells, fibroblasts, myofibroblasts, mesenchymal stem cells, pericytes, nerves and tumor-associated immune cells) [284]. The relative proportions of these cells are impacted by several factors, including inflammation and aging itself, and can determine the establishment of a pro-oncogenic or a tumor-suppressing microenvironment.

### 2.5.1. ECM remodeling

One of the most well-documented changes is tissue stiffening, which is associated with aging and cancer, among other pathologies [285, 286] (Fig. 3A). ECM stiffness regulates proliferation, migration and invasion of cancer cells, stemness, angiogenesis and the ability of tumor cells to evade immune system surveillance, and therefore governs cancer initiation and progression, metastasis and drug resistance [287]. Breast ECM stiffness increases with aging, and it is associated with enhanced invasiveness and aggressiveness of BC cells [282]. Moreover, matrix stiffness can promote epithelial-to-mesenchymal transition (EMT) in hepatocellular carcinoma, and patients with stiffer livers display a higher incidence of cancer recurrence [230].

Multiple transcription factors have been implicated in the regulation of cancer progression in response to matrix stiffness, including NF-κB, SOX2, β-catenin, YAP/TAZ, Snail and HIF1A [287]. Aging also affects ECM remodeling enzymes: the activity of the matrix metalloproteinases (MMP), which degrade ECM components, increases with age [288], while the production of ECM components and ECM-modifiers by aged fibroblasts decreases [289], thereby facilitating the invasion and metastasis of tumor cells [286]. For example, in a BC aging model, MMP-1 expression increased in the TME, and it is associated with enhanced local tumor growth and formation of brain metastases [290]. Similarly, in the skin, the increased age-related degradation of the ECM is a main determinant of melanoma progression [289].

**Figure 3.**
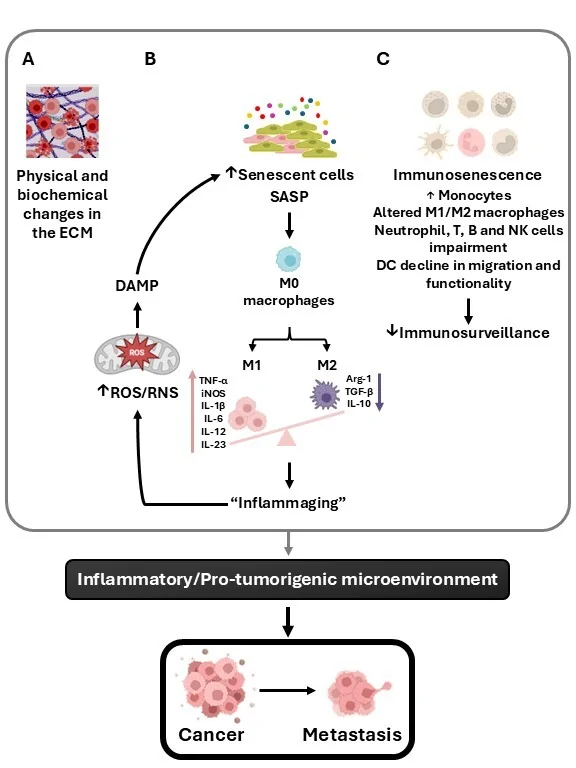
**Processes that occur during aging that promote cancer initiation and progression: ECM modifications and “inflammaging”**. (**A**) The aging microenvironment plays an important role in the initiation and progression of cancer, mostly due to physical changes in the extracellular matrix (ECM), facilitating the invasion and metastasis of tumor cells. (**B**) Senescent cells accumulate with aging and display alterations in the secretome (i.e. senescence-associated secretory phenotype, SASP), resulting in a chronic, low-grade inflammation (i.e. “inflammaging”) that can induce cancerous cell proliferation and tumor progression. SASP can transfer senescence to adjacent cells, perpetuating the inflammatory state. Inflammation promotes the generation of reactive oxygen/nitric species (ROS/RNS), inducing mutagenesis and epigenetic alterations. Reciprocally, dysfunctional mitochondria, with the production of damage-associated molecular pattern (DAMP), sustain ROS/RSN production and inflammation, promoting senescence. (**C**) Immunosenescence represents the decline in immune function with aging, it leads to a decrease in the immunosurveillance and weak anti-tumor immune responses and is associated with “inflammaging”.

The impact of aging on the composition of the microenvironment has been elegantly illustrated by Li *et al*., who studied mammary epithelial and stromal changes in mice [291]. They observed a decrease in the proportion of fibroblasts with age, along with a reduced expression of ECM-related genes, suggesting an impaired ability to maintain stromal architecture. On the contrary, vascular endothelial cells exhibited a drastic age-related increase, accompanied by upregulation of genes associated with cytokines involved in the modulation of the immune microenvironment, and downregulation of genes associated with cell-cell junctions. These changes indicate altered endothelial-immune interaction, increased vascular permeability, and the establishment of an inflammatory microenvironment conducive to malignant cell proliferation and metastasis. Similarly, the intra-tumoral stroma of BC patients increases with age and can influence the response to therapy [292].

### 2.5.2. Inflammaging and immune microenvironment alterations

The ECM serves not only as a structural scaffold but also as a source of critical biochemical (e.g., HGF, IGFS, FGFs, TGF-β) and biomechanical cues that dictate cell growth, survival, migration and differentiation, and regulate immune function and vascular development. Given that aging is accompanied by a chronic, low-grade inflammatory state-termed “inflammaging - characterized by elevated concentrations of mediators such as interleukin (IL)-6, tumor necrosis factor-α and C-reactive protein [293], one way aging can facilitate carcinogenesis is by inducing an inflammatory microenvironment (Fig. 3B).

Although in some instances immune cells can mediate immune surveillance and trigger anti-tumor immune responses, their permanent activation could lead to chronic inflammation, enabling and promoting the progression of several types of solid cancers [46, 294], for example PC [295].

Key cellular players in inflammaging include monocytes and macrophages. With age, there is an increase in circulating CD16+ monocytes, and aged monocytes exhibit decreased efferocytosis, migration and phagocytosis, as well as impaired resolution of inflammation due to lower expression of specific receptors, and reduced type-I IFN production. Inflammaging also alters the balance between M1 (pro-inflammatory, anti-tumorigenic) and M2 (anti-inflammatory, pro-tumorigenic, pro-angiogenic) macrophages (Fig. 3B). While macrophages in healthy elderly hepatic and adipose tissue display a more pro-inflammatory M1 phenotype, in elderly lymphoid tissues, lung and muscle, the proportion of immunosuppressive M2 macrophages increases. Interestingly, M2 macrophages trigger angiogenesis *in vivo* [296], and their number correlates with neovascularization and poor prognosis in tumor models [297] and human cancers [298].

A study on elderly BC patients found that, compared to the younger control group, those aged over 80 had a poorer tumor-immune microenvironment (TIME) and higher infiltration of M2 macrophages [299], which have been shown to promote BC cells metastasis *in vitro* and *in vivo* by secreting chitinase 3-like protein 1 [300]. Aging also reduces macrophages expression of co-receptors and major histocompatibility complex class II molecules, impairing antigen-presenting capacity [301]. Moreover, aged macrophages display increased senescence-associated markers and production of inflammatory cytokines, while phagocytosis, immune resolution, autophagy and toll-like receptors (TLRs) expression are decreased [302], changes driven by a downregulation of MYC and USF1 transcription factors [303].

Also neutrophils, which regulate T cell function, display age-associated functional impairments such as reduced phagocytic activity, impaired chemotaxis [304] and dysfunction of TLRs [305]. These changes, together with altered macrophages, have been postulated to contribute to chronic low-grade inflammation, leading to dysregulated macrophage-mediated immunosuppression and progression of a wide range of ARDs, including cancer [304]. Several studies have linked senescent neutrophils to the development and progression of prostate, lung and breast cancers, while in turn cancer cells can promote neutrophil aging, creating a vicious cycle that fosters tumor growth and metastasis [306].

Chen *et al.* have recently illustrated the negative influence of TME on CD8+ T cells functionality and their role in tumor control [307]. Reduced CD8+ T cell infiltration and function in aged mice correlate with enhanced tumor growth, and transferring T cells from young mice fails to restore tumor control in aged mice due to rapid induction of T cell dysfunction. The authors describe a tumor-infiltrating age-associated dysfunctional T cell (TTAD) state that is functionally, transcriptionally and epigenetically different from canonical T cell exhaustion. Altered natural killer (NK) cell-dendritic cell (DC)-CD8+ T cell crosstalk in aged tumors promotes a TTAD state.

Aging is accompanied not only by changes in the number of T cells, but also in their metabolism and expression of receptors, which reduces their cytotoxicity and ability to kill tumor cells [306]. In addition, although T lymphocytes mainly exert anti-tumor effects, senescent T lymphocytes facilitate immune evasion of malignant cells [282].

The expression of the immunosuppressive immune checkpoint-associated molecules (e.g., Tim-3 and TIGIT), increases with age, further contributing to tumor initiation and progression [308].

Similarly, aging alters B cells phenotype and immune function, increasing the risk of cancer and autoimmune diseases [306]. Aging-associated B cells, which exhibit a B-cell lymphoma phenotype, accumulate in older mice and humans, and particularly in elderly individuals predisposed to B-cell lymphoma [309].

NK cells, main effectors of innate immunity, display receptors that recognize stress-induced autologous proteins on cancer cells, making them critical in cancer immunity [310]. Aging alters NK cells' number, phenotype and function (e.g., lower receptor expression, diminished cytokine secretion capacity) [311, 312], contributing to an increased susceptibility to cancer in older adults. Remarkably, Peng *et al.* reported a marked reduction in cells expressing NKp30 or NKp46 in patients with pancreatic, gastric and CRC [313].

Also DCs exhibit age-related decline in migration, antigen-presentation, phagocytosis, chemotaxis and IFN production [314], reducing immune system’s ability to recognize and eliminate cancer cells. Accordingly, the use of a DC hyperactivator leads to the correction of these age-related defects and restores anti-tumor activity of DCs in elderly [315].

Inflammation might drive carcinogenesis via oxidative stress and the generation of reactive oxygen species (ROS) and reactive nitric species (RNS) [316]. These reactive species damage proteins, lipids and nucleic acid, constituting the damage-associated molecular pattern (DAMP), recognized as “non-self” by the immune system, thereby perpetuating inflammation [317]. Aging further exacerbates oxidative stress due to mitochondrial dysfunction, dysregulated NOX activity and other free-radical generating sources, leading to the formulation of the oxidative stress theory of aging [318] (Fig. 3B), supported by preclinical and observational studies linking high levels of ROS/RNS to many ARDs and cancer [319, 320].

### 2.5.3. Senescence and ECM

Senescent cells, which resist apoptosis, progressively accumulate in most tissues with aging [321], contributing to the loss of tissue homeostasis [322], a key hallmark of aging [322], and to the overall aging process [181]. This accumulation of noncancerous senescent cells in aged tissues might negatively affect tumor suppression and therefore exert pro-cancerous effects. For instance, senescent cancer-associated fibroblasts (senCAF) have been identified in mouse and human breast tumors, where they secrete ECM components that blunt the cytotoxicity of the NK cells, thereby promoting tumor growth. Interestingly, senCAFs are present in HER2+, ER+ and triple-negative BC, as well as in ductal carcinoma *in situ* (DCIS), where they predict tumor recurrence [323]. Beyond fibroblasts, senescent adipocytes also contribute to the TME. In the aging mammary gland, hypertrophic adipocytes promote immune cell recruitment and white adipose tissue inflammation, which increases local estrogen production. This mechanism may partially explain the higher incidence of hormone-dependent BC in older woman, despite declining systemic estrogen levels [324].

As already mentioned, a central feature of senescent cells is SASP, that can promote precancerous cell proliferation [325] and reshape the TME, including its immune component, facilitating immune evasion [283, 326] (Fig. 3B). Within the TIME, SASP can exert dual effects: on one end, it stimulates anti-tumor immunity, enhancing therapies efficacy; on the other it fosters immunosuppressive cell infiltration, thereby dampening immune surveillance [283]. In addition, SASP impairs stem cell functions, promotes cellular transdifferentiation, remodels the ECM and spreads senescence to surrounding cells, perpetuating the inflammaging [293] (Fig. 3B) and the systemic chronic inflammation [327]. The reduced efficiency of senescent cells clearance with age, further exacerbates this burden, while accelerates senescence of immune cells (i.e., macrophages) [328] weakening immune defense in older organisms [329], providing a more permissive environment for tumor growth.

SASP is both promoted by [330] and capable of [331] promoting DDR, establishing a feed-forward loop that reinforces senescence. Due to this persistent proinflammatory state [332], senescent cells have been causally linked to many ARDs [43, 333, 334].

Immunosenescence, a specialized form of senescence [335] affecting adaptive [283, 336, 337] and innate immune cells [283, 338, 339], further shapes the aged TME. It is associated with inflammaging, SASP production, increased glycolysis and ROS and reduced T-cell production [293, 340], contributing to cancer susceptibility [306] (Fig. 3C). Epigenetic regulation (e.g., DNA methylation, histone modifications, microRNA regulation) drives much of this immunosenescence [341]. Similarly, chronic SASP-driven low-grade inflammation suppresses immune cell function, further aggravating immune deterioration [342]. Also, telomere attrition and reduced telomerase activity [343], together with antigenic stimulation and thymic involution [306], can contribute to lymphocyte senescence and functional decline. The consequences on cancer progression are profound: impaired immune surveillance allows metastatic outgrowth at distant sites [344], while immunosenescence in the TME may foster tumorigenesis and reduce the effectiveness of immunotherapy [345].

### 2.6. Circadian system

The circadian system controls daily (~24 h) physiological and behavioral rhythms, enabling organisms to synchronize internal physiological and metabolic processes with environmental periodic cues. This temporal regulation optimizes adaptation by activating or pausing specific processes at specific times of the day [346]. Circadian control extends beyond core clock genes (e.g., *CLOCK*, *PER*, *CRY*, *BMAL1*, *REV-ERB*) to many clock-controlled genes, including genes implicated in cell cycle, DNA damage repair, important metabolic processes and oxidative stress (Fig. 4). As reviewed by Gaucher *et al*. [347], accumulating evidence suggests a complex bidirectional interplay between the circadian clock and the cell cycle. Indeed, the transcriptional core clock machinery modulates key cell cycle regulators (e.g., WEE1, P21, P16, MYC), while several cell cycle proteins exhibit rhythmic oscillations and, in turn, influence the phase and amplitude of circadian rhythms. An aging-related defective expression of circadian clock genes may impair regulation of these downstream pathways, diminishing cells susceptibility to apoptosis, increasing genomic instability and potentially inducing carcinogenesis [348, 349] (Fig. 4).

A dysfunctional circadian system has been proposed as a link between aging and cancer [350]. Aging modifies circadian system by reducing entrainment capacity, decreasing the amplitude of endogenous rhythms and modifying their phases [351]. *Bmal1* knock-out mice exhibit premature aging phenotypes [352], although this may partly reflect the pleiotropic functions of *Bmal1* beyond circadian regulation. Similarly, loss of sirtuin 1 (SIRT1), which regulates circadian clock components by epigenetic mechanisms, results in premature aging and aging-related circadian phenotypes [353]. In the same line, the suprachiasmatic nucleus of aged mice displays lower levels of SIRT1 [354]. Noteworthy, SIRT1 dysregulation has also been linked to tumorigenesis [350].

**Figure 4.**
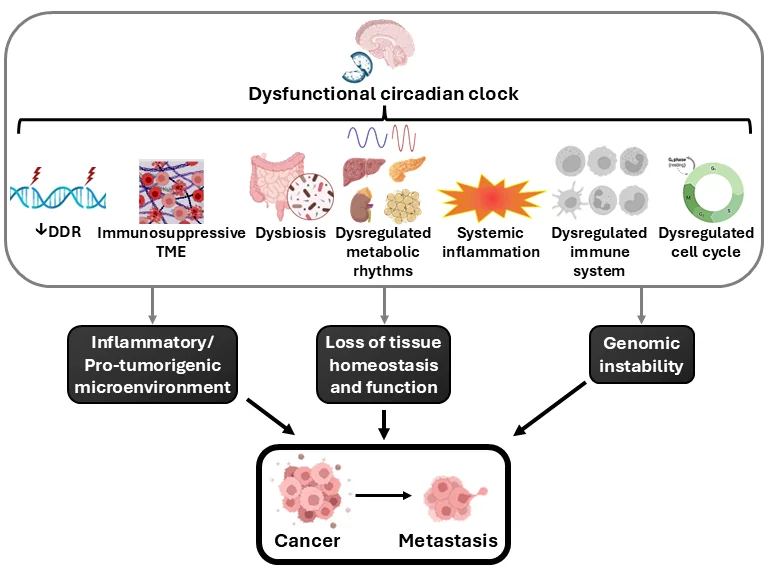
**Age-induced dysregulation of circadian clock promotes a permissive/favoring milieu for carcinogenesis, loss of tissue homeostasis and genomic instability**. Aging alters the circadian clock by disrupting the entrainment and decreasing the amplitude and modifying the phases of the endogenous rhythms. This dysregulation promotes an immunosuppressive tumor microenvironment (TME), which enhances the metastatic potential of cancer cells and results in altered metabolic rhythms that may lead to continuous activation of pro-oncogenic pathways, impaired DNA damage response (DDR) and enhanced genomic instability. Circadian misalignment may alter enteral microbiota leading to acceleration of aging and metabolic diseases, which in turn may trigger cancer development. Disturbances of the circadian clock have been also associated with systemic inflammation, dysregulation of immune systems and of the cell cycle, leading to an uncontrolled cell proliferation.

Circadian system regulates innate and adaptive immunity, two functions altered during aging and tumorigenesis [355] (Fig. 4). Aging abolishes diurnal innate immune responses and is accompanied by a loss of circadian gene transcription, a reduction in chromatin accessibility and a decrease in the diurnal expression of Kruppel-like factor 4 in macrophages [356]. Recently, it has been reported that circadian regulation of DCs trafficking to the tumor-draining lymph nodes dictates a rhythmic anti-tumor CD8+ T cell response, influencing melanoma growth; remarkably, the initial time-of-day of tumor engraftment determines subsequent tumor size in murine cancer models [357].

The comprehensive review by Xuan *et al.* describes the interactions of circadian clock with TME [358], while the review by Huang *et al.* describes in depth the mechanisms and factors by which the circadian system governs tumor development and progression, and the immune system [359]. Finally, the review by Li *et al.* describes the functions of circadian rhythms in the TME across various stages of cancer development, progression and metastasis, and points out the specific role of aging, angiogenesis and inflammation in governing circadian rhythms within the TME [360] (Fig. 4).

Clock genes alter metabolic rhythms either directly, by regulating key metabolic enzymes expression, or indirectly, by interacting with oncogenic targets. However, their role in cancer appears to be highly tumor- and context-dependent [359]. Thus, while many preclinical studies suggest tumor-suppressing roles of the circadian clock, new studies also point to tumor-promoting functions in multiple cancer types [361]. Moreover, the clock components also exert functions not related to circadian regulation via protein-protein interactions with other pathways that are essential for carcinogenesis [361].

Chronic disruption of circadian rhythms by genetic, epigenetic and environmental factors (e.g., chronic jet lag, shift-work) heightens cancer susceptibility and disease severity [362], as well as the risk and severity of comorbidities that impact cancer therapy [361]. As reviewed recently by Su *et al.*, disrupted circadian metabolic rhythms may contribute to carcinogenesis by promoting several metabolic diseases such as obesity, type 2 diabetes mellitus, metabolic dysfunction-associated steatotic liver disease or systemic inflammation [363] (Fig. 4). Aberrant daily oscillation of metabolites may perpetuate oncogenic signaling pathways, impair DNA repair and enhance genomic instability, triggering carcinogenesis [364] (Fig. 4).

Interestingly, host circadian rhythms and gut microbiota are interrelated, constituting the “microbiome-circadian clock-axis”. Diet, antibiotics, stress or aging can alter microbiota composition, disrupting circadian functions of the gastrointestinal tract, leading to metabolic diseases [365]. Conversely, circadian misalignment can reshape the microbiota, accelerating aging and several metabolic diseases [366] (Fig. 4).

### 2.7. Endocannabinoid system

The endocannabinoid system (ECS) comprises endocannabinoids (mainly anandamide and 2-arachidonoylglycerol [2-AG]), their receptors (i.e., cannabinoid receptors, CB1 and CB2; G protein-coupled receptor 55, GPR55; transient receptor potential vanilloid 1; nuclear receptors such as peroxisome proliferator-activated receptor gamma) and the proteins involved in the transport, degradation and synthesis of cannabinoids, such as diacylglycerol lipase (DAGL) α and β, fatty acid amide hydrolase, monoacylglycerol lipase (MAGL), and N-acylphosphatidylethanolamine-selective phospholipase D [367].

The ECS modulates many important physiological processes, and therefore its dysregulation has been linked to several ARDs, including cancer [368, 369] (Fig. 5). These conditions share common underlying mechanisms such as chronic inflammation, telomere shortening, SASP-driven inflammaging and immunosenescence. Preclinical studies suggest that the ECS contributes to healthy aging by regulating immune and inflammatory responses, suppressing oxidative stress, repressing ROS/RNS and stabilizing circadian rhythms [370]. They also improve quality of life (QoL) and general well-being in human trials [371]. Consistent with this, *Caenorhabditis elegans* studies showed that cannabinoids lengthen healthspan and lifespan, while attenuating the development of cellular senescence [371].

Age-dependent alterations of the ECS have been reported. Aged mice display a marked reduction in CB1 signaling, specifically in the hippocampus, as shown by a marked decrease in 2-AG and DAGLα protein levels [372]. Furthermore, genetic ablation of CB1 accelerates cognitive aging, induces neuronal loss and promotes hippocampal inflammation [373]. In addition, a big body of evidence implicated ECS dysregulation in the development and progression of age-related neurodegenerative diseases such as Alzheimer’s, Parkinson’s and Huntington’s disease [374].

As previously mentioned, endocannabinoid signaling is involved in the control of several cancer hallmarks, such as cell proliferation and migration, apoptosis, angiogenesis, tumor invasiveness and metastasis [375]. Consequently, a dysfunctional ECS may induce cancer development by fostering physiological conditions that facilitate the proliferation and migration of cancer cells [369] (Fig. 5). However, their effects appear highly context-dependent, varying with type of cancer, specific genetic mutations and the TME [376]. In this context, it has been shown that cannabinoids can impact the TME in several tumor models by modulating immune infiltration, angiogenic factors, metalloproteinases and non-metalloproteinases and oxidative stress [377] (Fig. 5). For instance, cannabinoids hamper the production of angiogenic factors such as angiopoietin-2 and hypoxia-induced vascular endothelial growth factor (VEGF) and prevent angiogenesis. Recently, it has been reported that enhanced levels of endogenous 2-AG, achieved by pharmacological inhibition of MAGL, elicited a marked reduction of VEGF expression in lung cancer cells that resulted in lower migration and tube formation when HUVECs were incubated with the conditioned medium from these cancer cells [378]. Similarly, cannabinoids modulated immune infiltration and downregulated MMP-2 [379].

Noteworthy, all the immune cells express cannabinoid receptors, positioning the ECS as a key regulator of immune homeostasis and a potential gatekeeper of immune function [369]. ECS modulates the activity of NK cells [380], which are essential in the removal of senescent cells, modulation of inflammation and initiation of the adaptive immune response [381]. Xiang *et al.* reported that MAGL deficiency contributes to lipid accumulation, macrophage activation, CD8+ T cell inhibition and tumor progression in several murine cancer models and suggested that MAGL-CB2 axis in macrophages could be a promising approach for cancer treatment. In addition, they showed that the expression of macrophage MAGL is decreased in cancer tissues and positively correlated with the survival of CRC patients [382].

Taken together, these findings support a close relation between ECS, immunosenescence and aging, suggesting that cannabinoids may hold therapeutic potential in immune disorders that are associated with various ARDs, including cancer [383].

**Figure 5.**
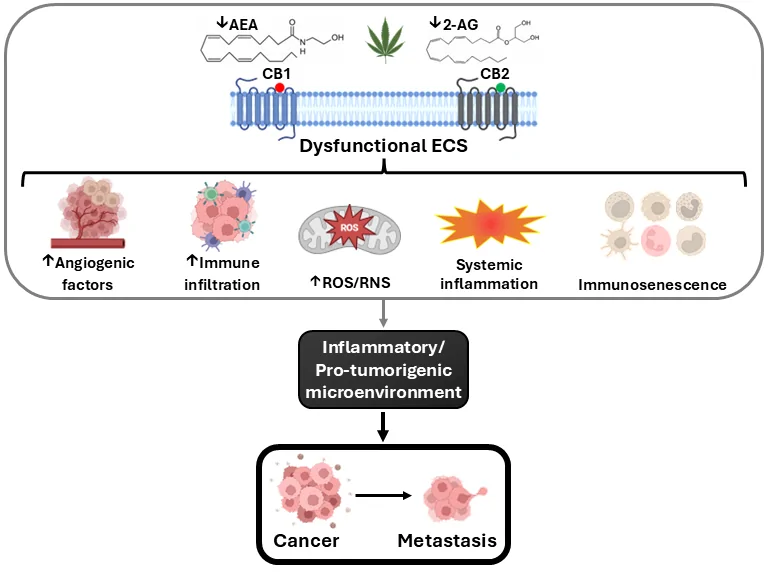
**Age-induced dysregulation of endocannabinoid system promotes a permissive/favoring milieu for carcinogenesis**. Aging blunts endocannabinoid signaling. The aged endocannabinoid system (ECS) fosters physiological conditions that facilitate the proliferation and migration of cancer cells, including enhanced secretion of angiogenic factors and MMPs, increased infiltration of immunosuppressive cells, high production of ROS/RNS, systemic inflammation and immunosenescence.

### 2.8. Gut microbiome

The gut microbiota, defined as the community of microorganisms inhabiting the human gastrointestinal tract, plays a fundamental role in regulating host physiology and health. Its composition changes throughout life, peaking in diversity around the age of 65 and declining thereafter, more pronouncedly in subjects over 80 years old [384]. Aging is accompanied by a gradual decrease in beneficial gut microbes and an increase of pro-inflammatory taxa, which may lead to ARDs and premature mortality [385]. For instance, the abundance of pathogenic bacteria such as *Escherichia coli*, *Butyricimonas virosa*, *Ruminococcus bicirculans*, *Bacteroides fragilis* and *Streptococcus vestibularis* increases with age in CRC, while health-associated probiotics such as *Eubacterium eligens*, decreases. Moreover, the structure and composition of the gut microbiota differ between healthy individuals and CRC patients at different ages [386].

Age-related changes in the gut microbiome are shaped by both intrinsic factors (e.g., progressive physiological decline) and extrinsic influences (e.g., therapies, diet, psychosocial factors, environmental exposures and geographic location).

Similarly, the microbiome profile is influenced by diseases. Increasing evidence links the human microbiome to cancer: microbiomes within the gut, as well as in local tissue and even within tumors, can either promote or restrain tumor progression and modulate responses to oncological therapies [387] (Fig. 6). Among the trillions of organisms comprising the human microbiota, the International Agency for Research on Cancer (IARC) currently designates only 11 as directly carcinogenic to humans (Group 1 carcinogens) [388]. However, many others have been reported to indirectly cause cancer. A recent review by Kandalai *et al.* summarizes the diverse contributions of specific genera and species to the development and progression of several cancers [387].

**Figure 6.**
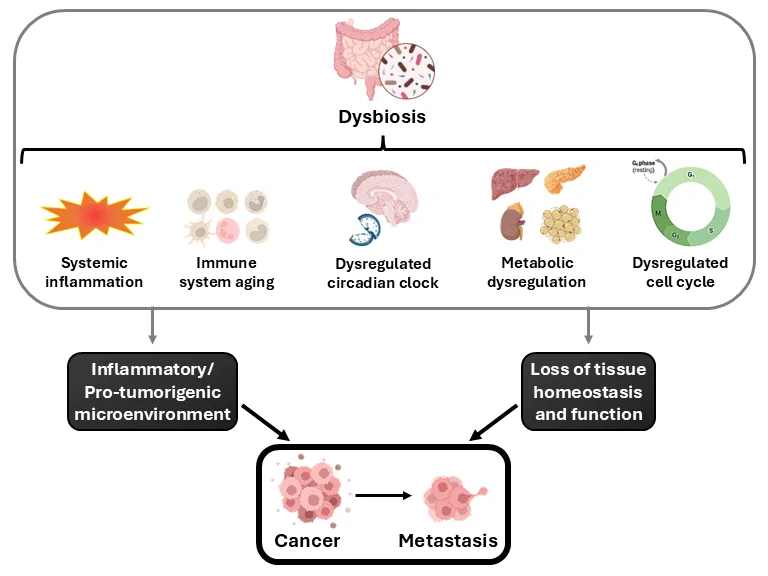
**Age-induced dysregulation of gut microbiome promotes a permissive/favoring milieu for carcinogenesis**. Aging is accompanied by dysbiosis, a gradual decline in the abundance of beneficial gut microbes and increase of pro-inflammatory microbes. Dysbiosis can promote systemic inflammation, aging of the hemato-immune system, disruption of the circadian clock, metabolic dysregulation and alterations in the cell cycle leading to cancer initiation and progression.

Age-related and disease-related decay of the gut microbiome of older individuals reflects overlapping but distinct processes [389]. Several studies suggest that alterations in the gut microbiota composition and function, known as dysbiosis, are linked to ARDs and may contribute to the aging process. Indeed, dysbiosis has been shown to affect systemic inflammation, immune function and metabolism [390], which are all hallmarks of aging (Fig. 6). In particular, dysbiosis accelerates hemato-immune aging by fostering a pro-inflammatory tissue environment, with the dominance of specific microbiota correlating with worse outcomes in hematologic malignancies [391]. Of note, centenarians often display a distinct gut microbiome composition with greater diversity and higher abundance of health-associated taxa such as *Akkermansia* and *Christensenellaceae* [392]. Conversely, non-healthy long-lived individuals tend to have higher *Streptococcus* abundance, linked to abnormal biological metabolism, whereas healthy longevity is associated with greater diversity and predominance of *Bacteroides* [393].

A recent study investigating the causal association between gut microbiota and biological age acceleration revealed that increased *Streptococcus* abundance can accelerate aging, while *Eubacterium* (*rectale group*), *Sellimonas*, *Actinomyces*, *Butyricimonas*, *Lachnospiraceae* (*FCS020 group*) had suggestive causal effects on aging acceleration, and *Lachnospira* showed potential protective effects by decelerating aging [394]. Notably, *Streptococcus* dysbiosis has been associated with several ARDs and cancers, including multiple myeloma (MM) [395], lung cancer [396], CRC [397] and gastric cancer [398]. Mechanistically, *Streptococcus* can enhance oxidative stress and induce chromatin remodeling [399], two processes implicated in both tumorigenesis and aging. Likewise, higher abundance of *Sellimonas* predicts increased risk of ER+ BC, while greater abundance of the class *Alphaproteobacteria* correlates with a lower risk of PC [400]. *Eubacterium rectale* could trigger CRC initiation by promoting inflammation [401], whereas higher levels of *Lachnospiraceae* species are inversely correlated with CRC risk, in part through IL-10-mediated mechanisms [402]. Similarly, *Alphaproteobacteria* abundance appears protective against PC [403]. The role of *Actinomyces* remains complex: although it seems to be associated with aging acceleration, some studies report a negative correlation with esophageal cancer [404], while others identify it, along with *Butyricimonas*, as enriched in patients with recurrent esophageal cancer, suggesting that *Butyricimonas spp*. may be a biomarker of postoperative recurrence [405].

## 3. Clinical trials and anti-aging/cancer therapies in older adults

Although older adults (over 65 years old) are at increased risk of chemotherapy toxicity [406], studies on cancer therapy in this population remain limited. Therefore, evidence-based recommendations in everyday clinical practice are still difficult to establish. Most preclinical studies use young animals, and clinical trials (CTs) frequently underrepresent older adults [407, 408]. Recruitment in this age is particularly challenging due to comorbidities and frailty. Furthermore, randomized CTs (RCTs) should be specifically tailored for the older population, including adapted assessments and patient-reported outcomes.

Given the rapid aging of the global population, agencies for health and cancer research have undertaken a multipronged strategy to improve the evidence-based practice for cancer treatment in older adults [409-411]. In this regard, in March 2022, the Food and Drug Administration (FDA) issued guidelines on the inclusion of older adults in cancer CTs [412], underlying the importance of understanding the benefit-risk profile of drugs also in older patients, to better inform treatment decisions.

Currently, 369 studies are retrieved after entering the terms “Cancer” and “Aging”, and filtering for “Older Adults (65+)” on ClinicalTrials.gov [413]. Of note, only 95 are specifically designed for older adults. Among them, 43 are completed; 11 are active, not recruiting; 18 are recruiting; 5 are not yet recruiting; 1 is enrolling by invitation; 3 have been terminated; 1 has been withdrawn; and 13 are on unknown status. Thirty-six of these CTs are observational, while 59 are interventional. Among them, 46 are randomized; 25 have supportive care as primary purpose, 11 have treatment as primary purpose and 8 are focused on prevention. Table 1 shows the main characteristics of the CTs listed in the following subsections.

### 3.1. CTs assessing the utility of the geriatric assessment

Geriatric oncologists face two major challenges: selecting specific patients’ treatments and balancing the extension of patients’ survival, while maintaining independence and QoL. To this end, the geriatric assessment (GA) is recommended to guide treatment choice in older patients [414-417]. A comprehensive GA (CGA) is a multidisciplinary evaluation encompassing functional status, comorbidity, polypharmacy, cognition, psychological status, social support and nutritional status [414, 418, 419], helping in a better estimation of the patient's overall fitness and therapy outcome. However, simplified screening tools are also available to identify patients most likely to benefit from a CGA [419, 420]. In addition, specific CGAs have been designed to predict chemotherapy side effects, including the Aging Research Group chemotoxicity calculator [421], the Chemotherapy Toxicity Tool and the Chemotherapy Risk Assessment Scale for High-Age Patients [422], the Index4 [423] or disease-specific calculators such as the Aging Research Group chemotoxicity calculator-breast cancer [424].

**Table 1:** **Characteristics of the CTs listed in Section 3**. Clinical trials and anti-aging/cancer therapies in older adults.

NCT Number (Status)	Conditions	Interventions	Phase	Allocation	Controlled	No. pts.	Age (years)	Primary outcomes	Ref.
**Geriatric assessment**									
**NCT01472094** **(Active, not recruiting)**	BC under chemotherapy	Observational	N/A	N/A	No	700	≥65	Develop a predictive model of clinical and biological predictors for grade 2-5 toxicity to adjuvant and neoadjuvant chemotherapy	[424]
**NCT04478916** **(Unknown)**	Hematologic malignancies; Solid tumor; Elderly patients	Observational	N/A	N/A	Yes	144	≥65	Number of patients received planned treatment and CGA evaluated with improved QoL	[425]
**NCT03154671** **(Completed)**	Cancer	GAM	N/A	R	Yes	351	≥70	QoL C30 Questionnaire Global QoL scale	[426]
**NCT02884375** **(Recruiting)**	Hematologic malignancies; Solid cancer	Observational	N/A	N/A	No	3000	≥70	Difference between initial oncologist treatment proposal and final treatment selected after GA	[427]
**NCT02517034** **(Active, not recruiting)**	Solid neoplasm	CGA; QoLA	N/A	R	Yes	600	≥65	Rate of grade 3-5 toxicity during chemotherapy	[429]
**NCT02054741** **(Completed)**	Adult solid neoplasm; Toxicity; Lymphoma	CGA; QoLA	N/A	R	Yes	733	≥70	QoLA (EQ-5D)	[430, 431]
**NCT05919641** **(Unknown)**	NSCLC; Stereotactic body radiotherapy; CGA	CGA (Observational)	N/A	N/A	Yes	130	≥70	QoLA (EQ-5D)	
**NCT03894917** **(Recruiting)**	HCC	CGA (Observational)	N/A	N/A	Yes	84	≥18	Characterize the change in disease and treatment patterns	
**NCT06040801** **(Recruiting)**	GIC	Frailty intervention measures	N/A	NR	Yes	138	≥65	Completion rate of chemotherapy; Grade and frequency of AEs and treatment-related toxicity; QoL	
**NCT04618809** **(Unknown)**	Metastatic gastric cancer	Didactic session; CGA; Fitness trackers for evaluation of functional status	N/A	N/A	No	100		Number of geriatric abnormalities (CGA); Percentage of treatment plan changes	
**N/A**	Solid malignancy at any stage	Cancer and Aging Research Group GA	N/A	N/A	No	96	≥75	Feasibility of implementing GA-guided interventions	[433]
**Physical exercise**									
**NCT04052126** **(Completed)**	Aged; 80 and Over; AML; Lymphoma,NHL; Fatigue; Exercise	Individualized PA program	N/A	N/A	No	43	≥65	Compliance rate	[436]
**N/A** **(Completed)**	Curable cancer under systemic treatment	12-week mixed exercise program (aerobic+resistance); Stretching program	N/A	R	Yes	20	65-85	Feasibility	[437]
**N/A**	Cancer under radiotherapy and/or chemotherapy w/o bone metastasis	12-week resistance exercise	N/A	R	Yes	240	≥60	Cancer-related fatigue measures	[439]
**NCT05424055** **(Completed)**	Cancer; Hospital acquired condition; Debility; Aging	Multicomponent exercise	N/A	R	Yes	58	≥65	Change in functional capacity; Change in cognitive function	[442]
**NCT05509751** **(Recruiting)**	Lung cancer; GIC; Genito-urinary cancer; BC; Gynecologic cancer; Lymphoma	GAM, exercise, health education; Online chair-based exercise combined with health education	N/A	R	Yes	60	≥65	Feasability; Acceptability of the intervention	[411]
**NCT03331406** **(Completed)**	Metastatic GIC; PA	PA program	N/A	N/A	No	20	≥18	Rate of accrual; Number of participants that adhered to PA program	[446]
**Nutritional interventions**									
**NCT04306562** **(Terminated)**	Sarcopenia; Cancer	Enteral protein supplementation	N/A	R	Yes	5	≥65	Postoperative complications	
**NCT00446888** **(Completed)**	Cancer	Standardized meals for diet stabilization; Forticare (DS); Product 4808	N/A	R	No	35	≥65	Muscle fractional synthetic rate of growth	
**NCT00000611** **(Completed)**	Postmenopausal women w/o prior BC	Fat-restricted diet w/o CR	N/A	R	Yes	48,835	50-79	Deaths from BC	[453, 459]
**NCT06496438** **(Recruiting)**	Depression; Anxiety disorder; Probiotics; GIC; Chemotherapy	Psychobiotics (DS); Placebo	N/A	R	Yes	270	18-85	Depression changes	
**NCT06819254** **(Not yet recruiting)**	Fatigue	Fisetin followed by Placebo; Placebo followed by Fisetin	4	R	Yes	60	≥65	Change in Pittsburgh Fatigability Scale	
**NCT04073381** **(Completed)**	Prehabilitation in surgical patients with abdominal cancer	Prehabilitation program	Early Phase 1	N/A	N/A	92	18-90	Length of hospital stay; Emergency room visits; Readmission; Mortality	[454]
**NCT04495751** **(Completed)**	Fatigue	Muscadine grape extract (DS); Placebo	Early Phase 1	R	Yes	64	≥65	Patient Reported Outcomes Measurement System; Fatigue 7a questionnaire	[455, 456]
**NCT04367493** **(Completed)**	Nutrition-related cancer; Aging	Chocolate (DS)	N/A	R	Yes	45	60-83	Change in nutritional status; Change in food ingestion; Change in body composition; Change in QoL	[458]
**NCT04713332** **Unknown**	Radiation-induced Injuries in patients with rectal cancer	Vitamin E (DS); Hydrogen rich water (DS); Placebo (DS)	3	R	Yes	60	≥18	Total blood count; Antioxidant enzymes; Oxidative stress markers	
**NCT00427193** **(Completed)**	Aging	CR; Control (*Ad libitum*)	N/A	R	Yes	238	21-50	Change in core body temperature; Change in resting metabolic rate	[462]
**NCT03595540** **(Completed)**	Cancer; BC; CRC	Prolon-FMD	N/A	N/A	N/A	90	≥18	Percentage of prescribed diet consumed and intake of any extra food; Quantification of FMD-emergent AEs	[464]
**NCT02970188** **(Unknown)**	Aging	TRF	1/2	R	Yes	12	55-79	Endothelium dependent dilation	[476]
**NCT03590847** **(Completed)**	Overweight	TRF	N/A	N/A	N/A	10	≥65	Adherence; Retention; Safety and tolerability; Pro-inflammatory markers	[479]
**N/A**	Multiple myeloma	TRF; Nordic walking training	N/A	N/A	Yes	40	≥60	Microbiota composition	[480]
**NCT02583269** **(Completed)**	Advanced malignant neoplasm; Metastatic malignant neoplasm; Unresectable malignant neoplasm	Laboratory biomarker analysis; Muscadine grape skin extract; QoLA	1	NR	No	24	≥18	Number of participants with DLT	[502]
**NCT01745263** **(Completed)**	Improve healthy ageing in seniors; Prevent disease at older age	Vitamin D3; Omega-3 fatty acids; Strength home exercise; Flexibility home exercise	3	R	Yes	2157	≥70	Incident non-vertebral fractures; Functional muscle decline; Blood pressure change; Cognitive decline; Rate of infections	[503-506]
**NCT04733534** **(Active, not recruiting)**	Frailty; Childhood cancer	Dasatinib+Quercetin; Fisetin	3	R	No	110	≥18	Change in walking speed; Senescent cell abundance in blood	
**NCT05724329** **(Active, not recruiting)**	Head and neck squamous cell carcinomas	Tislelizumab+Dasatinib+Quercetin (neoadjuvant); Surgery; Tislelizumab+Dasatinib+Quercetin (adjuvant)	Yes	Yes	N/A	18-80	2	Major pathological response	[508]
**Chronotherapy**									
**NCT04735939** **(Unknown)**	Glioma	Radiotherapy (Observational)	N/A	N/A	No	80	18-80	Survival time	
**NCT06845267** **(Not yet recruiting)**	Chronic fatigue; Cancer survivors	Chronotherapy; Control	N/A	R	Yes	200	≥18	Cancer-related fatigue	
**NCT02781792** **(Completed)**	Glioma; GM	Temozolomide; Functional assessment of cancer therapy-brain; ActTrust Condor instrument watch	2	R	No	42	≥18	Compliance with assigned administration time; Duration of response	[517]
**NCT04864405** **(Completed)**	BC	Morning administration of ET; Evening administration of ET	4	R	No	247	≥18	Endocrine toxicity and tolerability	[518]
**NCT01693861** **(Completed)**	mCRC	Observational	N/A	N/A	No	16	≥18	Effect of chemotherapy on urinary excretion of modified nucleosides	
**NCT06850766** **(Recruiting)**	IDH-Wildtype Glioblastoma; Glioblastoma	Morning administration of temozolomide; Evening administration of temozolomide	N/A	R	No	50	≥18	Adherence to temozolomide dose timing protocol	
**NCT06882174** **(Not yet recruiting)**	Metastatic NSCLC	Scheduling of the time of pembrolizumab infusions	2	R	Yes	58	≥18	Rate of deviation from scheduling intervention; Dropout rate	
**NCT06418139** **(Not yet recruiting)**	Non-Metastatic BC	Observational	N/A	N/A	No	450	18-100	Residual cancer burden class	
**NCT00852228** **(Unknown)**	mCRC; Liver metastases; Hepatic lesions	IV cetuximab; HAI chronomodulated chemotherapy; HAI conventional chemotherapy	2	NR	Yes	60	≥18	Incidence of complete macroscopic resections of unresectable liver metastases	[519]
**NCT04263948** **(Recruiting)**	Pancreas cancer	Picado system internet platform and connected objects	N/A	N/A	No	42		Toxicity-related emergency hospitalization rate; Rates and grades toxicity and early tumor responses	[550]
**NCT04374721** **(Unknown)**	Cushing syndrome; Adrenal insufficiency; Addison disease	Circadian gene expression evaluation	N/A	NR	Yes	44	18-80	CLOCK and ARNTL expression	
**NCT00519168** **(Completed)**	Advanced BC	Urine and saliva sample; Electrode sleep recorder; Phlebotomy (Observational)	N/A	N/A	Yes	123	45-75	Home and in-hospital polysomnography; Actigraphy; Self-report of sleep; Endocrine measures	[551]
**Epigenetic modifiers**									
**NCT03220347** **(Terminated)**	NHL; Neoplasms	CC-90010	1	N/A	N/A	139	≥18	AEs; DLT; Maximum tolerated dose	[534]
**NCT00404508** **(Completed)**	Refractory solid tumors	Hydralazine and magnesium valproate	2	NR	N/A	15	≥18	Clinical benefit; Safety	[535]
**NCT00532818** **(Unknown)**	Metastatic cervical cancer	Hydralazine and magnesium valproate; Placebo	3	R	Yes	143	≥18	Progression-free survival	[536]
**NCT02717884** **(Unknown)**	AML; Myelodysplastic syndrome	Tanylcypromine+All-trans retinoic acid+ Cytarabine	1/2	N/A	N/A	60	≥65	MTD determination of Tanylcypromine in combination with ATRA and with Cytarabine	[537]
**NCT01829503** **(Completed)**	AML	Decitabine and cytarabine; Supportive care	2	N/A	N/A	44	≥60	Number of participants with best clinical response; Proportion of participants with clinical response	[538]
**NCT05958719** **(Recruiting)**	Peripheral T-cell lymphoma; Epigenetic repression	Chidamide; Azacitidine; Liposomal mitoxantrone; Prednisone	2	NR	N/A	37	≥18	ORR; Number of participants with treatment-related AEs	
**NCT02395627** **(Terminated)**	Stage IV ER+ BC	Tamoxifen; Vorinostat; Pembrolizumab	2	R	No	38	≥18	ORR; Number of participants with Treatment-related AE	[540]
**NCT03903458** **(Unknown)**	Malignant melanoma	Tinostamustine+Nivolumab	1	N/A	No	21	≥18	Safety and DLT	[541]
**NCT04407741** **(Recruiting)**	Solid tumor; Lymphoma	SHR2554+SHR1701; SHR1701	1/2	R	Yes	100	18-70	Median amount of time subject survives without disease progression	[542, 543]
**Cannabinoids**									
**NCT01812603/ NCT01812616** **(Completed)**	GM	Sativex+Temozolomide	1/2	N/A	N/A	21	≥18	Incidence of AEs	[548]
**NCT04428203** **(Completed)**	Recurrent prostate cancer	Epidiolex oral liquid product	1	NR	N/A	21	≥18	Number of participants with DLT	[547]
**NCT05629702** **(Recruiting)**	Glioblastoma; Brain tumor	Nabiximols; Temozolomide; Nabiximols-matched placebo	2	R	Yes	120	≥16	Overall survival time	
**NCT05525455** **(Terminated)**	Advanced solid tumor	TT-816; TT-816+PD-1 inhibitor	1/2	NR	N/A	9	≥18	ORR; Incidence of AEs and serious AEs; Incidence and nature of DLT; MTD; Recommended Phase 2 dose	
**NCT02255292** **(Unknown)**	Solid tumor	Cannabidiol	2	N/A	N/A	60	≥18	ORR	
**NCT06601218** **(Recruiting)**	Cancer; Cancer pain	Oral cannabis low THC; Oral cannabis high THC; Oral cannabis THC/CBD; Placebo comparator	1	R	Yes	80	≥18	Percent of participants that withdraw from the study or are removed due to AEs	
**NCT02423239** **(Unknown)**	HCC; Pancreatic cancer	Dexanabinol; Sorafenib; Nab-paclitaxel	1	NR	No	112	≥18	MTD of dexanabinol+standard chemotherapies; Number of AEs	
**NCT06266611** **(Recruiting)**	Sleep; Anxiety; Depression	fsCBD Cannabidiol; bsCBD Cannabidiol; Placebo	2	R	Yes	185	≥25	Pain interference; Pain intensity; Sleep disturbance; Fatigue	
**NCT05246670** **(Active, not recruiting)**	CIPN; Hematopoietic and lymphoid cell neoplasm; Malignant solid neoplasm	Palmidrol; Placebo; QoLA	2	R	Yes	88	≥18	Mean change in QoLA	
**NCT05822362** **(Recruiting)**	Mild cognitive impairment	Cannabidiol; Placebo	2	R	Yes	236	55-85	Neurocognitive function; Biomarkers of Alzheimer’s disease progression	
**NCT05188404** **(Completed)**	Cannabis use	Choice of cannabis product (Observational)	N/A	N/A	Yes	326		Change in patient global impression of change; Change in FACT-Cog; Balance task	

AEs: Adverse events; AML: Acute myeloid leukemia; BC: Breast cancer; CGA: Comprehensive geriatric assessment; CIPN: Chemotherapy-induced peripheral neuropathy; CR: Calorie restriction; CRC: Colorectal cancer; DLT: Dose-limiting toxicity; DS: Dietary supplement; ET: Endocrine therapy; FMD: Fasting mimicking diet; GA: Geriatric assessment; GAM: Geriatric assessment and management; GIC: Gastrointestinal cancer; GM: Glioma multiforme; HCC: Hepatocellular carcinoma; mCRC: Metastatic CRC; MTD: Maximum tolerated dose; N/A: Not applicable; No.: Number; NHL: Non-Hodgkin lymphoma; NSCLC: Non-small cell lung cancer; ORR: Overall response rate; PA: Physical activity; pts.: Patients; QoL: Quality of life; QoLA: Quality of life assessment; Ref.: Reference; TRF: Time-restricted feeding.

Several trials have tested the importance of GA and CGA in relation to cancer therapy in older adults [424-428]. They incorporate both physiological and cognitive/behavioral effects to build a predictive model for chemotherapy toxicity. Two large RCTs demonstrated that identifying and addressing vulnerabilities through GA reduced chemotherapy-related toxicities by 10%-20% and increased treatment adherence [429, 430]. The results of the RCT done by Li et *al.* (NCT02517034) in ≥ 65 years patients with solid malignancies showed that GA-driven intervention (GAIN) significantly reduced grade ≥ 3 chemotherapy-related toxicities [429]. Similarly, the RCT NCT02054741 in patients over 70 years with incurable solid tumors, including stage III & IV lung cancer, or lymphoma, showed that providing a GA summary and management recommendations significantly improved treatment tolerability [430, 431].

Additional CTs are currently ongoing to assess the impact of CGA or GAIN on the management of old and frail oncologic patients, including NCT05919641 (localized non-small cell lung cancer), NCT04478916 (onco-haematological patients candidates for complex therapies) [425], NCT03894917 (hepatocellular carcinoma), NCT06040801 (frail patients with gastric, biliary and pancreatic cancer receiving palliative chemotherapy) and NCT04618809 (metastatic gastric cancer) [432]. Furthermore, a recent prospective pilot study demonstrated the feasibility of implementing GAIN among hospitalized older adults with cancer to identify vulnerabilities and to guide the interventions in the inpatient oncology setting [433].

### 3.2. CTs assessing physical exercise

Several studies are focusing on lifestyle interventions aimed at reducing side effects of cancer therapy associated with both aging and cancer. For example, different trials point out that physical exercise during or after radiotherapy and chemotherapy decreased molecular aging and tumor markers, while improving patients’ fitness and QoL [434-436]. Evidence indicates that physical exercise can reduce pain, toxicity, fatigue, and improve mental state, QoL and mobility, as well as adherence to treatment [437-439]. In line with these findings, an integrative literature review showed that combined aerobic and resistance exercise protocols improved the functional capacity of older cancer patients [440]. Similarly, a recent systematic review confirmed the effectiveness of physical exercise, as part of a holistic management of the patient, for preventing disability, improving QoL, and partially reducing all-cause mortality [441]. Furthermore, the RCT NCT05424055 showed that an individualized multicomponent exercise program (twice daily for five days) in hospitalized older adults with cancer (mean age 74.4 years) was not only feasible, but also improved functional abilities and reduced fatigue in comparison to standard hospital care [442]. Moreover, physical exercise on chemotherapy treatment days is safe and potentially effective in reducing neuropathy and maintaining physical well-being [443, 444]. Additional benefits include decreased hospital admissions and shorter hospital stays [445].

Particular attention must be given to frail older adults undergoing cancer treatment, given that they are at heightened risk of adverse outcomes. Nonetheless, older cancer patients usually report low levels of physical activity, and poor adherence to recommendations provided by health centers. Consequently, trials are also exploring how to better involve older people with cancer in structured exercise programs (NCT04052126 [436], NCT05509751 [411], NCT03331406 [446]). Of note is the recent systematic review and meta-analysis showing that the potential harms of exercise during systemic cancer treatment are uncertain, and current evidence is insufficient to establish robust risk-benefit profiles for the application of structured exercise in this population [447].

Therefore, larger controlled RCTs are needed to evaluate the impact of physical activity on cancer progression, treatment-related side effects, post-operative recovery, long-term outcomes and QoL in older cancer patients, while accounting for their comorbidities and functional decline. These studies, which should incorporate patient-reported outcomes, will provide evidence-based guidance for a safe inclusion of physical activity in the holistic management of older cancer patients. In the field of prevention, large and long-term studies in young/adult subjects are warranted to evaluate whether different physical activity regimens promote healthy aging and thereby reduce cancer risk.

### 3.3. CTs assessing nutritional interventions

Epigenetic alterations are involved in both cancer and aging, and diet and nutrition are known modulators of epigenetic mechanisms [448]. Thus, epigenetics could provide a link between nutrition and cancer therapy in older adults [449]. Despite the general use of dietary supplements after cancer diagnosis, there is no consensus regarding their recommendation by medical authorities, including the World Cancer Research Fund and the American Cancer Society [450]. Therefore, different studies are testing the impact of modified food regimens and dietary supplements on aging, cancer incidence and survival, chemotherapy-induced toxicity, hospitalization rates and infections (e.g., NCT04306562, NCT00446888, NCT00000611 [451-453], NCT06496438, NCT0681 9254, NCT04073381 [454], NCT04495751 [455, 456]) [457]. In this context, it has been proved that, in older cancer patients receiving palliative care, the consumption of chocolate with high cocoa content improved nutritional status and physical/mental functionality (NCT04367493) [458]. Antioxidant interventions are also being explored, such as hydrogen-rich water or vitamin E, which may mitigate radiotherapy-related side effects in old colon cancer patients (NCT04713332).

Dietary fat reduction has been specifically evaluated in the WHI DM trial (NCT00000611), which randomized 48,835 postmenopausal women (50-79 years) without prior BC to a low-fat dietary intervention or to a usual diet [459]. Although the 8.5-year intervention phase showed a nonsignificant trend toward reduced, in an extended follow-up (19.6 years), BC incidence and mortality displayed a statistically significant reduction in all-cause mortality following BC, as well as in BC-specific mortality [460].

Caloric restriction (CR, sustained 10-40% reduction in daily energy intake), without malnutrition, has been shown to slow aging [461, 462] and extend lifespan. Therefore, CR may reduce the risk of many ARDs, including cancer [463]. In addition, CR, along with other nutritional interventions (e.g., short-term fasting, fasting-mimicking diet [FMD]), seems also to have beneficial effects in combination with cancer therapy [464-468]. However, in non-obese older patients, CR is rarely feasible due to frailty and comorbidities [469], and it may cause lean mass and bone density loss, damped immune system function and increased risk of malnutrition.

Intermittent fasting (IF), which alternates unrestricted feeding with shorter periods of restricted intake, and short-term fasting or FMD (5-day low calorie intervention, typically plant-based, designed to mimic the metabolic effects of a water-only fast while still providing necessary nutrients) are considered potential CR-mimicking lifestyle strategies in humans [464-467, 470]. Their metabolic benefits are likely achieved by impacting (i) modifiable lifestyle behaviors (e.g. sleep), (ii) gut microbiome, (iii) immune responses, (iv) circulating cytokines/adipokines and (v) circadian biology [467, 471].

A particular form of IF is time-restricted feeding (TRF) in animals, or time-restricted eating (TRE) in humans, which involves consuming normal daily calories amount within a limited time window (3-12 h) and fasting during the rest of the day [471]. Common TRE protocols include the 16/8, 18/6 and 20/4 schedules, which result in an 8 h, 6 h and 4 h eating window, respectively [472]. Importantly, the eating window should be aligned with the circadian rhythms (i.e., eating during active daytime hours) to optimize metabolic processes [473]. Notably, TRF maximizes the physiological benefits of CR, while minimizing its adverse effects in older adults, given that, unlike all other forms of IF, it does not require caloric reduction [470]. This feature may also facilitate the long-term adherence to this eating pattern [474]. Indeed, a pilot study with 10 overweight, sedentary older adults, instructed to fast for approximately 16 h per day for 4 weeks, showed a high level of adherence, few reported adverse events (AEs) and clinically meaningful improvements in walking speed and QoL [475]. Similarly, a randomized, controlled pilot study of short-term TRE (eating < 8 h per day for 6 weeks) in healthy, non-obese midlife and older adults demonstrated that this approach is safe and well-tolerated, and associated with high adherence, without impacting lean mass, bone density or nutrient intake [476]. Importantly, TRE exerts tumor-suppressive effects in healthy individuals and is feasible and acceptable by cancer patients, improves QoL and may have oncological benefits [477]. In this line, it has been reported that elderly individuals with the shortest fasting duration (≤ 7.5 h) display higher risks of both cancer and other-cause mortality, and that fasting duration of 11.49 h correlates with the lowest mortality risk [478]. Of relevance, a recent pilot study in overweight older adults suggests that TRE could reduce inflammation and oxidative stress [479]. Furthermore, a 10/14 TRE regimen combined with 6-week Nordic walking training program was able to change the gut microbiota of MM patients, which was predominantly represented by the phyla *Firmicutes*, *Actinobacteria*, *Verrucomicrobia*, *Proteobacteria* and *Bacteroidetes*, toward the pattern of the control healthy group [480]. Collectively, these findings suggest that TRE could be a promising therapeutic approach for the management of older cancer patients.

In the context of cancer prevention, larger CTs, with longer follow-up, using CR, FMD, IF or TRE should be performed to evaluate the potential of these interventions to promote healthy aging and eventually decrease the risk of cancer development in the elderly. These studies could generate evidence-based data that would allow health authorities to develop prevention programs to reduce the economic and health burden due to cancer.

### 3.4. CTs assessing calorie restriction mimetics

Calorie restriction mimetics (CRMs) are both bioactive food components and non-dietary substances (e.g., metformin, hydroxycitrate, spermidine, rapamycin, aspirin and natural polyphenols such as resveratrol) that replicate the biochemical and signaling pathways triggered by CR. CRMs elicit antitumor activity enhancing anticancer immunosurveillance and modulating energy and nutrient-sensing pathways, chaperone-mediated autophagy and epigenetic factors [481-483]. They also enhance the therapeutic efficacy of chemo-immunotherapies (e.g. immune checkpoint inhibitors targeting PD-1) [484]. CRMs are well tolerated, with metformin and aspirin showing the strongest evidence for reducing cancer risk in a selected group of patients.

Currently, there are 46 CTs registered in ClinicalTrials.gov evaluating CRMs. Although all of them include older adults, only 16 are specifically designed for subjects aged over 65, and only 1 was conducted in cancer settings (Table 2). This CT (NCT02642094) tested the effect of sirolimus (mTOR inhibitor) in human breast tissue. While no effect was observed on basal cell population, luminal cell population was reduced, particularly in postmenopausal patients. Most importantly, it also significantly reduced prognostic biomarkers associated with BC progression from ductal carcinoma *in situ* to invasive BC (i.e., p16INK4A, COX-2, Ki67), as well as markers of the SASP, thereby conceivably preventing early BC progression [485].

**Table 2:** Clinical trials studying calorie restriction mimetics (CRMs) in older adults.

NCT Number	Status	Conditions	Interventions	Age (years)	Phase	Ref.
**NCT06459310**	Recruiting	Metformin; Aging	Metformin hydrochloride tablet; Placebo	18-65	2	
**NCT02432287**	Completed	Aging	Metformin; Placebo	≥60	4	
**NCT02308228**	Completed	Aging	Progressive resistance training; Metformin	≥65	1	[552]
**NCT03861767**	Terminated	Aging	Metformin ER; Placebo	≥18	3	[553]
**NCT03072485**	Completed	Aging	Sirolimus; Metformin; Diclofenac	≥55	1	
**NCT03451006**	Terminated	Aging; Inflammation; Frailty	Metformin; Placebo	≥60	2	
**NCT03309007**	Completed	Prediabetes; Aging	Metformin; Placebo	30-70	3	
**NCT03713801**	Completed	Aging; Vaccine response impaired	Metformin; Placebo	63-90	1	
**NCT03996538**	Completed	Aging; Age-related immunodeficiency; Vaccine response impaired	Metformin hydrochloride ER Tablets; Influenza vaccine	≥65	1	[554]
**NCT01765946**	Completed	IR; Prediabetes; Aging; Inflammation	Metformin; Placebo	40-75	4	[555]
**NCT04264897**	Completed	Aging; Insulin sensitivity; Chronic disease; Mitochondria; IR	Metformin; Placebo	40-75	3	[556]
**NCT04994561**	Withdrawn	Aging	Metformin+Dasatinib+Bio-Quercetin+Bio-Fisetin+Glucosamine+Nicotinamide riboside+Trans-Resveratrol	≥65	1	
**NCT06463743**	Not yet recruiting	Multiple sclerosis	Metformin; Placebo	55-75	2	
**NCT03107884**	Active, not recruiting	Muscle atrophy; IR	Metformin (Bed rest); Placebo (Bed rest); Metformin (2 week run-in only); Placebo (2 week run-in only)	≥60	1	[557, 558]
**NCT04221750**	Active, not recruiting	Frailty; Sarcopenic obesity; Aging	Metformin hydrochloride; Placebo; Lifestyle therapy	65-85	3	[559]
**NCT05893849**	Unknown	Aging	Clinical probes	≥60	Ob	[560]
**NCT02745886**	Unknown	Overweight; Aging	Metformin; CR	18-60	4	
**NCT04375657**	Recruiting	Epigenetic aging; Immunosenescence	Somatropin+Metformin+Deidroepiandrosterone; Metformin+ Deidroepiandrosterone	40-80	2	
**NCT06999343**	Not yet recruiting	COPD	Metformin 850Mg Tab; Placebo	40-75	3	
**NCT06445569**	Completed	Prediabetes; Aging	KH-1 (DS); Placebo	≥50	NA	
**NCT06550271**	Completed	Aging	Rapamycin	40-120	Ob	
**NCT02874924**	Completed	Aging	Rapamycin; Placebo	70-95	2	[561]
**NCT04488601**	Completed	Aging	Rapamycin; Placebo	50-85	2	[562]
**NCT04742777**	Recruiting	Aging	Rapamycin	70-95	2	
**NCT01649960**	Completed	Aging; CAD	Rapamycin	≥60	1	[563]
**NCT06658093**	Not yet recruiting	Aging	Rapamycin; Everolimus; Placebo	65-90	1	
**NCT02642094**	Terminated	Breast cancer	Rapamycin	18-80	2	[485]
**NCT06727305**	Not yet recruiting	Aging	Sirolimus; Everolimus	65-80	1/2	
**NCT05949658**	Recruiting	Aging	Sirolimus; Everolimus	55-89	1	
**NCT05835999**	Recruiting	Aging; IR	Everolimus; Placebo	18-80	2	
**NCT00891696**	Completed	Sarcopenia	Everolimus; Physical training	18-85	2	[564]
**NCT04608448**	Completed	Aging; Epigenetics; Inflammatory mediators	Rapamycin topical ointment; Placebo	65-95	1	
**NCT05237687**	Recruiting	Aging	Sirolimus	65-80	2	
**NCT04284397**	Recruiting	Aging	Low dose Aspirin; Control	≥18	1	
**NCT02523274**	Completed	Aging	Resveratrol; Placebo; Exercise	≥65	2	[565]
**NCT05981053**	Completed	Aging; Healthy aging; Sarcopenia; Obesity	Wine enriched with Resveratrol	40-80	Ob	[566]
**NCT05500742**	Unknown	Aging	Coenzyme Q10 (DS); Selenium (DS); Resveratrol (DS); TA-65 MD (DS); Placebo-1; Placebo-2	65-80	NA	[567]
**NCT02909699**	Completed	Aging	Resveratrol	65-99	Ob	
**NCT00823381**	Completed	Obesity; Metabolic syndrome; Diabetes; Aging	Resveratrol (DS); Placebo; CR	35-70	NA	[568]
**NCT01842399**	Terminated	Vascular resistance; Hypertension	Resveratrol (DS); Placebo	≥50	1/2	
**NCT01126229**	Completed	Memory	Placebo; Low dose Resveratrol; High dose Resveratrol	≥65	1	[569]
**NCT00996229**	Unknown	Healthy	CR; Omega-3 (fish oil capsules); Resveratrol (DS); Placebo	50-80	3	[570]
**NCT02095873**	Completed	Glucose Intolerance; Aortic stiffness; Vasodilation	Glyoxalase 1 inducer (DS); Placebo	18-80	1/2	[571]
**NCT06585865**	Recruiting	Sarcopenia; Anabolic resistance	Resistance training; Resveratrol (DS); Placebo	≥60	NA	
**NCT02123121**	Completed	Mitochondrial function; Physical function	Resveratrol 1000 mg/day; Resveratrol 1500 mg/day; Vegetable cellulose (Placebo)	≥65	2	
**NCT02001831**	Unknown	Physical disability	DS: ω-3 PUFAs+Vitamin D3+Resveratrol	≥70	NA	[572]

DS: Dietary supplement; ER: Extended-release; IR: Insulin resistance; KH-1: Nutraceutical comprised of spermidine, spermidine derivatives and probiotics; NA: Not applicable. Ob: Observational; Ref.: Reference. Source: ClinicalTrials.gov, accessed on 2025/07/02.

### 3.4.1. CTs assessing senotherapeutics

Senotherapeutics are a particular class of CRMs, which either selectively kill senescent cells (senolytics) or suppress the SASP (senomorphics) [486-488]. Since cellular senescence is implicated in aging-related phenotypes [489] and in the onset of ARDs, in particular cancer [323, 490-492], pharmacological interventions targeting senescent cells have been proposed to delay age-driven tumorigenesis [492-494].

The first senolytics identified were dasatinib (a pan-inhibitor of tyrosine kinase receptors) and quercetin (a flavonoid targeting Bcl-2 family members), which combination reduced senescent cells burden and ARDs in progeroid mice [495]. Since then, additional classes of senolytics have been reported, including kinase inhibitors (e.g., saracatinib, nintedanib), natural compounds (e.g. fisetin), Bcl-2 family inhibitors (e.g. navitoclax), p53 binding inhibitors (e.g., UBX0101, FOXO4-DRI, P22077/P5091), heat shock protein 90 (HSP90) inhibitors (e.g., 17AAG, 17-DMAG), Na+/K+ ATPase pumps inhibitors (e.g., ouabain, digitoxin), galactose modified prodrugs (e.g., duocarmycin, gemcitabine) and histone deacetylase (HDAC) inhibitors (e.g. panobinostat) [487, 491, 496].

In cancer therapy, senolytics have been proven to reduce chemo- and radiotherapy adverse effects, and to rejuvenate chemotherapy-induced aged tissues [497, 498]. Currently, the combination of dasatinib and quercetin is being investigated in adults who survived childhood cancer and present an accelerated aging phenotype (NCT04733534). Table 3 shows a list of the interventional CTs testing quercetin and/or fisetin in the context of cancer and/or aging. Remarkably, only 5 CTs were specifically conducted in patients aged ≥ 65 years.

However, first-generation senolytics, largely derived from drug repurposing, exhibit limited efficacy, underscoring the need for next-generation, tissue- and disease-specific senolytic strategies. Promising approaches include cell therapy and immunological strategies, such as antibody-drug conjugates, CAR-T cells or vaccines [496].

Senomorphics, also known as senostatics or SASP inhibitors, work by disrupting the connection between proinflammatory, proapoptotic senescent cells and disease, without acting directly on senescent cells. Senomorphics are inhibitors of ATM, p38 MAPK, JAK/STAT, mitochondrial complex 1- or 4-related and the NF-κB and mTOR pathways, and they include rapamycin, metformin, curcumin and ellagic acid, among others. Other senomorphics inhibit HSP90, modulate NAD+/NADH metabolism or neutralize SASP factors or their receptors [499]. Unlike senolytics, senomorphics require continuous administration because they suppress SASP activity but do not eliminate senescent cells [500].

**Table 3:** Interventional clinical trials studying quercetin and fisetin, two natural senolytics, in the context of cancer or aging.

NCT Number	Status	Conditions	Interventions	Age (years)	Phase	Ref.
**Cancer**						
**NCT02989129**	Withdrawn	Polyneuropathies and other disorders of the peripheral nervous system; Chemotherapy- induced neuropathic pain	Quercetin	≥18	1	
**NCT05680662**	Unknown	BC	Quercetin+EGCG+Metformin+Zinc	18-70	1	
**NCT01732393**	Completed	Chemotherapy-induced oral mucositis	Quercetin; Placebo	15-40	1/2	[573]
**NCT04733534**	Recruiting	Frailty; Childhood cancer	Dasatinib+Quercetin; Fisetin	≥18	2	
**NCT03476330**	Active, not recruiting	Fanconi anemia; Squamous cell carcinoma	Quercetin (DS)	≥2	2	
**NCT01538316**	Unknown	Primary prevention of PC	Quercetin (DS); Genistein (DS); Placebo	18-65	N/A	
**NCT00003365**	Terminated	CRC	Curcumin (DS); Rutin (DS); Quercetin; Sulindac	≥18	N/A	
**NCT05724329**	Active, not recruiting	Head and neck squamous cell carcinomas	Tislelizumab+Dasatinib+Quercetin (neoadjuvant); Surgery; Tislelizumab+Dasatinib+Quercetin (adjuvant)	18-80	2	[508]
**NCT05456022**	Unknown	Tongue cancer	Quercetin; Doxorubicin	Any	2	
**NCT06615752**	Terminated	Metastatic castration-resistant PC	Green tea+Quercetin+Docetaxel; Placebo+Docetaxel	≥18	1/2	
**NCT01912820**	Completed	PC	Green tea extract (DS); Quercetin; Placebo; Surgery	40-75	1	[574]
**NCT06209229**	Completed	NSCLC	Sodium nucleinate (DS)	40-65	1	
**NCT06355037**	Recruiting	Triple-negative BC	Quercetin; Dasatinib; Taxane/Anthracycline/Eribulin mesylate/Vinorelbine/Capecitabine/Carboplatin/UTD1/Platinum	18-70	2	
**NCT06940297**	Recruiting	Recurrent MM; Refractory MM	Cyclophosphamide; Dasatinib; Fludarabine; Quercetin	≥18	2	
**NCT02195232**	Completed	Thromboembolism of Vein VTE in CRC; in Pancreatic cancer and in NSCLC	Isoquercetin	≥18	2/3	[575]
**NCT03493997**	Completed	PC	Radiotherapy+IAluril®+Laluril Soft Gels®; Radiotherapy	≥18	2	[576]
**NCT05651321**	Unknown	Follicular lymphoma	ω-3 fatty acids; Selenium; Garlic extract; Ellagic acid; Resveratrol+Quercetin; EGCG	≥18	2	
**NCT06819254**	Not yet recruiting	Fatigue in cancer survivors	Fisetin followed by Placebo; Placebo followed by Fisetin	≥65	4	
**NCT05595499**	Recruiting	Stage I-III BC	Fisetin; Placebo	≥60	2	[577]
**NCT06113016**	Recruiting	Stage I-III BC	Fisetin; Placebo	≥50	2	[578]
**NCT07025226**	Not yet recruiting	Glioma	Dasatinib; Fisetin; Fluorodopa F 18; Quercetin; Temozolomide	≥18	1	
**NCT01879878**	Unknown	Pancreatic Ductal Adenocarcinoma	Verum, broccoli sprout grain; Placebo	≥18	N/A	
**NCT02446795**	Unknown	Renal Cell Carcinoma; Kidney Cancer	Sunitinib; Isoquercetin; Placebo	≥18	1/2	
**NCT00455416**	Unknown	Follicular Lymphoma	Omega 3 fatty acids; L-Selenomethionine; Allicin; Ellagic acid; Resveratrol, Quercetin; Epigallocathechin gallate	≥18	2	
**Aging**						
**NCT04946383**	Unknown	Aging	Dasatinib+Quercetin	≥40	2	[579]
**NCT05422885**	Completed	Aging	Dasatinib; Quercetin	≥65	1/2	[580, 581]
**NCT05297032**	Terminated	Lipid metabolism; Aging	Quercetin phytosome (DS)	20-70	N/A	
**NCT05838560**	Recruiting	Premature aging; Schizophrenia; Treatment resistant depression	Dasatinib; Quercetin	≥50	2	
**NCT01376011**	Completed	Stroke; Problem of Aging	Quercetin (DS); Placebo	18-75	1	
**NCT04994561**	Withdrawn	Aging	Metformin+Dasatinib+Rapamycin+Bio-Quercetin+Bio-Fisetin+Glucosamine+Nicotinamide riboside+Trans-Resveratrol	≥65	1	
**NCT01752868**	Completed	Healthy	Curcumin+Fish oil+Resveratrol+Sesamin+Acetyl-L-carnitine+Lipoic acid+Green and black teas+Quercetin+Pomegranate+Cinnamon bark	40-60	N/A	
**NCT06133634**	Recruiting	Aging; Endothelial dysfunction; Arterial stiffness	Fisetin (DS); Placebo	≥65	1/2	
**NCT06399809**	Recruiting	Peripheral arterial disease; Aging; Peripheral vascular diseases; Walking; Difficulty; Claudication	Fisetin; Placebo	≥50	2	
**NCT03675724**	Enrolling by invitation	Frail elderly syndrome	Fisetin (DS); Placebo	≥70	2	
**NCT05838560**	Recruiting	Schizophrenia; Treatment Resistant Depression; Aging, Premature	Dasatinib; Quercetin	≥50	2	[582]
**NCT07000734**	Not yet recruiting	Aging; Cognitive decline; Older adults; Immune senescence; Motor function; Sedentary behaviors	Dasatinib; Quercetin; Vortioxetine; Risk managment	50-70	2	
**NCT06990256**	Not yet recruiting	Sleep disorder; Aging	Fisetin; Placebo; Urolithin A	45-70	N/A	

BC: Breast cancer; CRC: Colorectal cancer; DS: Dietary supplement; EGCG: Epigallocatechin gallate; MM: Multiple myeloma; NA: Not applicable; NR: Nonrandomized; NSCLC: Non-small cell lung cancer; PC: Prostate cancer; R: Randomized; Ref.: Reference. Source: ClinicalTrials.gov, 2025/07/02.

A recent work compared the effects of senolytics and of senomorphics in rescuing radiotherapy-induced frailty phenotype in a mouse model. Prolonged metformin administration delayed frailty and improved short-term memory, achieving benefits comparable to senolytic administration [501].

Among natural compounds, the muscadine grape extract (MGE) is under investigation for its anticancer properties. This extract is a mixture of senotherapeutic polyphenols (e.g., gallic acid, quercetin, cyanidin, delphinidin, ellagic acid) contained in the grapes of *Vitis rotundifolia*, and has been proven as an effective anticancer therapy in different *in vitro* cancer models and has been reported safe and well-tolerated in heavily pre-treated and older cancer patients (NCT02583269) [502]. Currently, the clinical trial NCT04495751 is investigating whether MGE improves fatigue in people over 70 years who have a history of treated cancer [455].

The DO-HEALTH trial, further investigated the geroprotective effects of vitamin D (2,000 IU per day) and/or omega-3 (1 g per day) and/or a home exercise program in generally healthy and active adults aged ≥ 70 years. Results showed that omega-3 supplementation alone reduced the rate of falls by 10% [503] and the rate of infections by 13% [504]. When combined, all three interventions showed a significant additive benefit on reducing pre-frailty by 39% [505] and incident invasive cancer by 61% [503] over a 3-year follow-up. Remarkably, a post *hoc* analysis on the effect of these treatments on four next-generation DNA methylation (DNAm) measures of biological aging (GrimAge, GrimAge2, DunedinPACE and PhenoAge) revealed that, over 3 years, omega-3 alone slowed the DNAm clocks GrimAge2, DunedinPACE and PhenoAge, and all three treatments had additive benefits on PhenoAge. These results support the notion that targeted nutritional strategies can have distinct epigenetic aging effects. Moreover, the observation that subjects with lower starting levels of omega-3 displayed larger epigenetic shifts further strengthens the use of personalized strategies [506]. These observations also endorse the geroscience hypothesis that slowing biological aging can contribute to prevent chronic diseases [507].

The preliminary results of a phase 2 CT (NCT05724329) combining anti-PD-1 therapy (tislelizumab) with dasatinib and quercetin in patients with head and neck squamous cell carcinoma show a favorable safety profile and therapeutic efficacy of the combination, with a reduction in AEs and alleviated immunosenescence [508].

As previously mentioned, nutritional interventions, which imply CR, are difficult to implement in older cancer patients, who very often present conditions such as frailty, sarcopenia and cachexia. CRMs are a feasible alternative, since they are well tolerated. While CR and CRMs show promising effects in anticancer therapy, strong evidence is still needed to support their integration in standard-of-care treatments [509]. This is particularly true in the case of older cancer patients, who are often polymedicated and thus at greater risk of adverse drug reactions. Therefore, large RCTs specifically designed for this population should be conducted to assess not only the feasibility of these interventions, but also the interactions of the CRMs with the most common medications taken by these patients. Once demonstrated the safety, RCTs should be conducted to evaluate the efficacy of these drugs in combination with standard cancer therapies.

### 3.5. CTs assessing chronotherapy

Over 80% of the FDA-approved drugs target molecules that display daily rhythms. Therefore, the timing of drug administration should be considered to enhance the efficacy and safety of anti-aging and oncological drugs acting on oscillating targets [510]. This concept constitutes the base of the so called “chronotherapy or circadian medicine” [511].

As reviewed by Acosta-Rodríguez *et al.*, chronotherapy in aging rests on three main observations: (i) aging is accompanied by a decline in circadian rhythms, (ii) aging-related pathways oscillate along the day and (iii) disruption of circadian rhythms results in metabolic disorders and reduce lifespan, whereas circadian rhythms restoration improves health and longevity [510]. Sulli *et al.* have proposed that restoring the balance of metabolic rhythms improves cancer therapies. Such restoration can be achieved by (i) drugging the clock by targeting core clock components, (ii) clocking the drug by optimizing the time of administration for optimal efficacy (chronotherapy), and (iii) training the clock by reinforcing daily rhythms through behavioral cycles (light exposure, sleep, physical exercise and feeding) [512]. A summary of these interventions can be found in the review by Zhu *et al.* [361]. Currently, there are 10 CTs assessing chronotherapy [513], 15 evaluating circadian dysregulation [514] and 2 studying clock genes dysregulation [515] in the context of cancer. To date, 6 CTs study circadian dysregulation in the context of aging [516], while no trial investigating chronotherapy and aging is currently registered in ClinicalTrials.gov.

In the RCT conducted by Damato *et al.* (NCT02781792), 35 glioma patients (mean age 56.31, 20-81) were randomized to receive temozolomide either before 10 a.m. or after 8 p.m. Compliance data proved the feasibility of timed temozolomide dosing, but no significant differences in AEs, QoL or survival between the groups were observed. However, the authors claimed that a larger study is required to validate the effect of chronotherapy on clinical efficacy [517].

Another RCT (NCT04864405) randomized 245 women (mean age 61.1) starting adjuvant endocrine therapy for BC to either morning or evening administration. There were no statistical differences in endocrine toxicity/tolerability measured by the change in total Functional Assessment of Cancer Therapy-Endocrine Subscale (FACT-ES) score from baseline to 12-weeks, nor in QoL [518].

The NCT00852228 CT was conducted in patients (median age 58, 33-76) with unresectable liver metastases (LM) from *wt KRAS* CRC to test whether hepatic artery infusion (HAI) with triplet chemotherapy and systemic cetuximab could increase the rate of conversion of previously unresectable LM to curative intent in previously treated patients. To that aim, 46 patients were assigned to conventional HAI and 24 to chronomodulated HAI. Objective response rates (ORR) were markedly higher, and the rate of early responses was twice as large on chronomodulated when compared with conventional delivery (38.9% *vs.* 19.6%) [519].

Chronoimmunotherapy has also emerged as a promising anticancer strategy. Recent studies demonstrated that patients with stage IV malignant melanoma or metastatic non-small-cell lung cancer receiving immune checkpoint inhibitors exhibit time-of-day dependent OS [520, 521].

Available data on possible administration-time differences in drug dynamics in the elderly is very sparse, although the chronopharmacologic mismatch is one of the main contributors to poor therapeutic outcomes and treatment-related AEs. Therefore, additional prospective studies are needed to fully characterize the chronodinamics and chronokinetics of commonly used anticancer drugs in the elderly. Such studies will clarify whether administration-time effects are amplified or attenuated with age in comparison with young subjects, allowing to exploit the potential of the chronotherapy already suggested in young/adult patients to reduce toxicity while enhancing efficacy in older cancer patients.

Evidence for the impact of time-of-day on radiotherapy outcomes is even more limited and often with inconsistent results [522]. Furthermore, not all the studies include older adults. Two prospective RCTs conducted in head and neck cancer patients showed that afternoon treatments were associated with faster progression to a higher grade of mucositis, while the median time to develop grade III/IV mucositis was significantly longer in morning-treated patients [523, 524]. Conversely, a prospective RCT in cervical cancer patients found higher rates of severe gastrointestinal mucositis in patients treated in the morning compared with those treated in the afternoon [525].

The scarcity of RCTs and the disparity of the results of the studies carried out to date make necessary studies conducted under much more standardized conditions, using similar sources of irradiation, time intervals for morning/afternoon groups, symptoms used as the primary endpoint, as well as the use of consensus guidelines to evaluate treatment outcome.

### 3.6. CTs assessing epigenetic modifiers

As noted above, epigenetic alterations during aging contribute to several ARDs, including cancer. Epigenetics is a reversible process that can be countered by environmental factors, diet (epigenetic diet or “epidiet”, e.g., epigallocatechin-3-gallate, curcumin, resveratrol, genistein, sulforaphane) and epigenetic drugs, also known as “epidrugs” (e.g., azacitidine, decitabine, vorinostat, romidepsin, belinostat, panobinostat, tazemetostat, enasidenib and ivosidenib). Most epidrugs act as inhibitors of DNA methyltransferases, histone demethylases, histone acetyltransferases, HDACs and protein arginine methyltransferases, although in some contexts they can also act as activators [526, 527].

Epigenetic therapy impacts energy metabolism and induces cell differentiation, cell cycle arrest and cell death, thereby influencing multiple genes and proteins [527]. When used alone or in combination with chemotherapy, oncometabolites or immunotherapy, epidrugs enhance anti-tumor activity, reduce drug resistance and stimulate the host immune response in several hematological and solid tumors (e.g., breast, skin, colon, prostate) [528]. Some of them have already been approved by the FDA for different cancers and ARDs treatments, while others are in different phases of CTs [529, 530]. Currently, there are 192 ongoing or completed CTs assessing epidrugs in the context of cancer, of which 174 include older adults. Remarkably, none of them are specifically designed for older adults [531].

Several preclinical and clinical studies have shown the potential of epidrugs, epidiets and its combination in cancer prevention and therapy [532, 533]. For instance, the CT NCT03220347 tested CC-90010, a reversible and orally active BET inhibitor, in patients with advanced or unresectable solid tumors or relapsed/refractory diffuse large B-cell lymphoma (R/R-DLBCL). CC-90010 monotherapy displayed antitumor activity, with an ORR of 13.0% in patients with R/R-DLBC and a clinical benefit rate (CBR) of 31.7% in patients with advanced solid tumors, while toxicities were generally manageable [534]. Similarly, the CT NCT00404508 assessing the response and toxicity of hydralazine and magnesium valproate in patients with solid tumors receiving chemotherapy showed clinical benefit in 80% of the patients. A reduction in global DNA methylation, HDAC activity and promoter demethylation was also observed, while treatment was well-tolerated, despite the fact that the study population was heavily pre-treated [535]. A RCT (NCT00532818) enrolling 143 patients with metastatic, persistent or recurrent cervical cancer without previous systemic treatment demonstrated improved PFS for epigenetic therapy with hydralazine and valproate plus standard cisplatin+topotecan over cisplatin+topotecan [536].

The CT NCT02717884 is one of the few CTs specifically designed to test epidrugs in elderly patients (median age 75 years). It evaluates the efficacy of tranylcypromine (a first-generation histone demethylase LSD1 [KDM1A] inhibitor) in combination with ATRA and cytarabine in elderly, non-fit AML patients resistant to prior hypomethylating agent (HMA) treatment. The combination was feasible, even at the highest tranylcypromine dose. While the ORR was modest, 48% of the patients achieved partial remission or stable disease with this 3-drug combination, indicating limited cross-resistance with prior azanucleosides treatment [537]. The phase 2 study NCT01829503 is another trial conducted specifically in older adults. It was designed to determine the efficacy of an induction regimen using decitabine as an epigenetic primer followed by cytarabine in the treatment of 44 older patients (median age 76 years) with newly diagnosed AML. Complete response was achieved by 26 patients (66.7% of evaluable patients). Fourteen of 21 (66.7%) patients with adverse cytogenetics achieved a complete response. The 4- and 8-week mortality rates were 2.3% and 9.1%, respectively, with median OS of 10.7 months. There were no treatment-related mortalities. The authors suggest that epigenetic priming with decitabine followed by cytarabine should be considered as first-line option for older AML patients [538]. The ongoing CT NCT05958719 investigates the ORR of a new combination therapy called CAMP (chidamide, azacitidine, liposomal mitoxantrone and prednisone) as first-line therapy for untreated angioimmunoblastic T-cell lymphoma (AITL). It compares CAMP's safety and efficacy to standard treatments. Younger patients (≤ 70) receive the full CAMP regimen, while older patients receive a modified version (CAMP-light).

The combined use of epidrugs with immune checkpoint inhibitors (e.g., atezolizmab, avelumab, cemiplimab, durvalumb, ipilimumab, nivolumab, pembrolizmab) provides a novel and promising option for cancer treatment [529, 539]. For instance, a RCT (NCT02395627) in patients with therapy resistant ER+ BC tested the safety and response rate of the combination of a HDAC inhibitor (vorinostat), an anti-estrogen (tamoxifen) and a PD-1 inhibitor (pembrolizumab). Among evaluable patients who received all three agents, 3.7% achieved a complete response, with a CBR of 18.5% and an ORR of 3.7% [540]. Similarly, the NCT03903458 trial tested the combination of tinostamustine (EDO-S101) and the anti-PD-1 monoclonal antibody nivolumab in 17 patients with refractory, locally advanced or metastatic melanoma. This treatment resulted in 54% disease stabilization and 23% confirmed partial responses [541]. The CT NCT04407741 evaluates the EZH2 inhibitor SHR2554 in combination with the anti-PD-L1/TGFβ antibody SHR1701 in patients with advanced or metastatic solid tumors and relapsed/refractory B-cell lymphomas. In the efficacy-evaluable population, ORR was 57.7%, including 2 complete responses achieved by patients with metastatic renal cell carcinoma and classical Hodgkin lymphoma (cHL). Out of 14 cHL-evaluable patients, ORR was 100% and the complete response rate was 7.1%. [542, 543].

**Table 4:** Interventional clinical trials studying epidrugs in the context of cancer and/or aging.

NCT Number	Status	Conditions	Interventions	Age (years)	Phase	Ref.
**Cancer**						
**NCT01200004**	Terminated	Advanced cancers	Azacitidine; Lenalidomide; *Grifola frondosa*	≥13	1	
**NCT05178693**	Recruiting	Neuroendocrine tumors	ASTX727; Lutathera	18-99	1	[583]
**NCT02395627**	Terminated	Stage IV ER+ BC	Tamoxifen; Vorinostat; Pembrolizumab	≥18	2	[540]
**NCT03220477**	Active, not recruiting	NSCLC	Pembrolizumab; Guadecitabine; Mocetinostat	≥18	1	
**NCT01534598**	Completed	Neoplasms	FdCyd+THU	≥18	1	[584]
**NCT03220347**	Terminated	NHL; Neoplasms	CC-90010	≥18	1	[534, 585]
**NCT00404508**	Completed	Refractory solid tumors	Hydralazine+Magnesium valproate	≥18	2	[535]
**NCT04257448**	Unknown	Metastatic PDAC	Romidepsin; Azacytidine; Nab-Paclitaxel; Gemcitabine; Durvalumab; Lenalidomide capsule	≥18	1/2	[586]
**NCT04648826**	Withdrawn	Sarcomas; Melanomas; Germ cell tumors; Epithelial malignancies (excluding lung and renal cell carcinomas); Pulmonary metastases	Bintrafusp alfa; Azacitidine	≥18	1/2	
**NCT01700569**	Terminated	Grade IV astrocytoma; Glioblastoma	Temozolomide; folinic acid; High voltage radiation therapy	18-70	1	[587]
**NCT03612739**	Withdrawn	AML; MDS	NKR-2 CAR-T cells+5-azacytidine	≥18	1	
**NCT02374099**	Terminated	BC	CC-486; Fulvestrant	≥18	2	[588]
**NCT02664181**	Completed	NSCLC	Nivolumab; Decitabine; THU	≥18	2	
**NCT00533299**	Unknown	Ovarian cancer	Hydralazine+Magnesium valproate; Placebo	≥18	3	
**NCT01194908**	Terminated	BC	Decitabine; LBH589; Tamoxifen	≥18	1/2	
**NCT03903458**	Unknown	Malignant melanoma	Tinostamustine	≥18	1	[541]
**NCT04611711**	Unknown	Digestive system tumors resistant to PD-1 inhibitors	Decitabine+TQB2450 injection; Decitabine+TQB2450 injection+Anlotinib	≥18	1/2	
**NCT04965129**	Recruiting	Lung cancer	Fish oil; Placebo	20-90	N/A	
**NCT05680662**	Unknown	BC	Quercetin+EGCG+Metformin+Zinc	18-70	1	
**NCT00978432**	Terminated	DLBCL	RAD001; LBH589; RAD001+LBH589	≥18	2	
**NCT01121757**	Terminated	Follicular lymphoma; Marginal zone lymphoma	Azacitidine; Lenalidomide	≥18	2	
**NCT03682029**	Active, not recruiting	MDS; CML; Cytopenia	Vitamin C (DS); Placebo	≥18	N/A	
**NCT04190056**	Terminated	Stage IV BC	Pembrolizumab; Tamoxifen; Vorinostat	≥18	2	
**NCT00404326**	Completed	Cervical cancer	Hydralazine+Magnesium valproate	18-70	2	
**NCT01743560**	Completed	ER+ advanced BC	RAD001; Exemestane	≥18	4	
**NCT06694454**	Not yet recruiting	NSCLC	Azacytidine; Carboplatin; Paclitaxel; Durvalumab; Cisplatin; Gemcitabine; Pemetrexed	18-120	1/2	
**NCT00532818**	Unknown	Metastatic cervical cancer	Hydralazine+Magnesium valproate; Placebo	≥18	3	[536]
**NCT03765229**	Completed	Melanoma	Entinostat; Pembrolizumab	18-99	2	
**NCT04173585**	Completed	AML	Bortezomib; Gemtuzumab ozogamicin	18-100	2	
**NCT04355858**	Unknown	BC	SHR7390; Famitinib; SHR3162, Pyrotinib; Capecitabine; SHR1210; Everolimus; Nab- Paclitaxel; SHR2554; SHR3680; SHR6390; SHR1701; SERD; AI; VEGFi	18-75	2	[589]
**NCT02497404**	Completed	Acute leukemia erythroblastic; MDS	5-Azacytidine; Fludarabine; Melphalan; Alemtuzumab; Total body irradiation	≥18	2	
**NCT06714357**	Recruiting	Metastatic CRC	Irinotecan; Panitumumab; VPA	≥18	2	
**NCT00395655**	Terminated	Locally advanced BC	Hydralazine+Magnesium valproate	≥18	2	[590]
**NCT02717884**	Unknown	AML; MDS	Tranylcypromine; All-trans retinoic acid; Cytarabine	≥18	1/2	[537]
**NCT02951156**	Terminated	DLBCL	Avelumab; Utomilumab; Rituximab; Azacitidine; Bendamustine; Gemcitabine; Oxaliplatin	≥18	3	[591]
**NCT01016990**	Unknown	Leukemia; Lymphoma	VPA	≥18	2	
**NCT01607645**	Terminated	Leukemia; Refractory anemia with excess blasts	Decitabine; Idarubicin; Cytarabine	≥18	2	
**NCT02546986**	Active, not recruiting	NSCLC	CC-486; Pembrolizumab; Placebo	≥18	2	[592]
**NCT01729845**	Completed	Previously treated MDS; AML	Cytarabine; Decitabine; Etoposide; Mitoxantrone hydrochloride	≥18	1/2	[593]
**NCT02159820**	Unknown	Primary malignant neoplasm of ovary; FIGO stages II to IV	Decitabine; Paclitaxel+Carboplatin	18-80	2/3	
**NCT02846935**	Completed	T-cell lymphoma; Aggressive B-cell lymphoma; NHL; Indolent B-cell lymphoma	Decitabine; THU	≥18	1	
**NCT04407741**	Recruiting	Solid tumor; Lymphoma	SHR2554+SHR1701; SHR1701	18-70	1/2	[542, 543]
**NCT02847000**	Completed	Metastatic pancreatic adenocarcinoma	THU; Decitabine	≥18	1	
**NCT03366116**	Recruiting	Neoplasms; Solid tumors	5-aza-4'-Thio-2'-Deoxycytidine	18-120	1	[594]
**NCT02423057**	Suspended	Neoplasms; Solid tumors	4'-Thio-2'-Deoxycytidine	18-120	1	
**NCT00359606**	Completed	Neoplasms	FdCyd; THU	≥18	1	
**NCT00978250**	Completed	Head and neck neoplasms; Lung neoplasms; Urinary bladder neoplasms; Breast neoplasms	FdCyd+THU	18-120	2	
**NCT04741945**	Recruiting	Preleukemia; Preleukemic Anemia; Myelodysplastic neoplasm; Cytopenia	Metformin	≥18	2	
**NCT04553393**	Unknown	Refractory or relapsed aggressive r/r B-NHL with huge tumor burden	Chidamide; Decitabine; Chidamide+Decitabine; Decitabine-primed Tandem CAR19/20 engineered T cells	16-65	1/2	
**NCT02959437**	Terminated	Solid tumors; advanced malignancies; metastatic cancer	Azacitidine; Pembrolizumab; Epacadostat; INCB057643; Pembrolizumab; Epacadostat; INCB059872	≥18	1/2	[595]
**NCT01935947**	Terminated	Recurrent NSCLC; Stage IIIA NSCLC; Stage IIIB NSCLC;	Azacitidine; Azacitidine; Docetaxel; Entinostat; Gemcitabine hydrochloride; Irinotecan hydrochloride; Pemetrexed disodium; Laboratory biomarker analysis	≥18	2	
**NCT03903458**	Unknown	Malignant melanoma	Tinostamustine	≥18	1	
**NCT02518958**	Completed	Malignant solid tumor; Lymphoma	RRx-001; Drug: Nivolumab	≥18	1	
**NCT00925132**	Terminated	Metastatic melanoma	Temozolomide+Decitabine+Panobinostat	≥18	1/2	[596]
**NCT02900560**	Terminated	Epithelial ovarian cancer	CC-486; Pembrolizumab	≥18	2	
**NCT04896073**	Completed	Pancreatic adenosquamous carcinoma	Minnelide	≥18	2	[597]
**NCT06646809**	Recruiting	CRC	Alpha-ketoglutarate; Placebo	≥45	3	
**NCT06454448**	Recruiting	Metastatic pancreatic cancer	Adebrelimab+Decitabine	18-72	1/2	
**NCT05958719**	Recruiting	PTCL; Epigenetic repression	Chidamide; Azacitidine; Liposomal mitoxantrone; Prednisone	≥18	2	
**NCT01933061**	Withdrawn	Metastatic melanoma	Abraxane; Abraxane+CC-486	≥18	2	
**NCT01834248**	Completed	AML; Alkylating agent-related AML; CML; MDS; Refractory anemia with excess blasts in transformation	DEC-205/NY-ESO-1 fusion protein CDX-1401; Decitabine; Poly ICLC	≥18	1	
**NCT01265953**	Completed	Prostate cancer prevention	Sulforaphane-rich broccoli sprout extract capsules; Gelatin capsule containing microcrystalline cellulose	≥21	NA	[598]
**NCT03417427**	Unknown	AML	Decitabine+Ara-C; Ara-C	14-60	2	
**NCT02724202**	Unknown	Metastatic colon cancer	Curcumin; 5-flurorouracil	≥18	1	
**NCT02786875**	Unknown	BC	High level Vitamin D; normal level Vitamin D	30-74	3	[599]
**NCT00005639**	Completed	Lymphoma; Small intestine cancer; Unspecified adult solid tumor	Azacitidine injection; Sodium phenylbutyrate	18-120	1	
**NCT06930118**	Recruiting	Advanced CRC in the third-line treatment and beyond; pMMR/MSS advanced CRC	Chidamide+Regorafenib+Iparomlimab/Tuvonralimab	≥18	2	
**NCT02250326**	Completed	NSCLC	*nab*-paclitaxel IV; CC-486; Duravalumab	≥18	2	[600]
**NCT04541277**	Unknown	AML in relapse or refractory; AML in unfit elderly; AML with positive minimal residual disease	Tislelizumab+Decitabine+Azacitidine	≥18	2	[601]
**NCT03240211**	Recruiting	PTCL; Cutaneous T-cell lymphoma	Pembrolizumab; Pralatrexate; Decitabine	18-90	1	
**NCT02452970**	Terminated	Cholangiocarcinoma	RRx-001; Gemcitabine+Cisplatin	≥18	2	
**NCT02705469**	Completed	mCRPC	ZEN003694	≥18	1	
**NCT05896813**	Unknown	Newly diagnosed PTCL	CHOP+Chidamide	18-80	NA	
**NCT05497453**	Terminated	Hepatocellular carcinoma; Solid tumors	OTX-2002; TKI o; TKI two; Immune checkpoint inhibitor	≥18	1/2	[602]
**NCT02711956**	Completed	mCRPC	ZEN003694; Enzalutamide	≥18	1/2	
**NCT03900871**	Unknown	Esophageal squamous cell carcinoma	Acetylsalicylic acid	18-70	1	
**NCT01301820**	Completed	AML	Azacitidine; Lenalidomide	60-75	2	[603]
**NCT04831710**	Unknown	Angioimmunoblastic T-cell lymphoma	Sintilimab; Chidamide	18-75	2	
**NCT04414969**	Unknown	Early-stage extranodal natural killer/T-cell lymphoma	Anti-PD-1 antibody+Peg-Asparaginase+Chidamide	18-75	2	[604]
**NCT03999723**	Recruiting	MDS; AML; CML	Vitamin C (DS); Placebo (DS)	≥18	2	
**NCT01829503**	Completed	AML	Decitabine+Cytarabine; Supportive care	≥60	2	[538]
**NCT02489929**	Unknown	Myeloid Leukemia; MDS	Azacytidine	18-85	NA	
**NCT06705686**	Not yet recruiting	mCRPC	(R)-9bMS	≥18	1	
**NCT03206047**	Completed	Platinum-resistant fallopian tube carcinoma; Platinum-resistant ovarian carcinoma; Platinum-resistant primary peritoneal carcinoma; Recurrent fallopian tube carcinoma; Recurrent ovarian carcinoma; Recurrent primary peritoneal carcinoma	Atezolizumab; DEC-205/NY-ESO-1 Fusion Protein CDX-1401; Guadecitabine; Poly ICLC	≥18	1/2	
**NCT05772728**	Unknown	T-Cell lymphoma	Cidapenem+Azacitidine+Mitoxantrone liposome (CAM) regimen	18-75	N/A	
**NCT03709550**	Withdrawn	CRPC; Metastatic prostate carcinoma in soft tissue; Prostate carcinoma metastatic in bone	Decitabine; Enzalutamide	≥18	1/2	
**NCT01358045**	Completed	Basal cell carcinoma	Diclofenac; Diclofenac+Calcitriol; Calcitriol	≥18	2	
**NCT02085408**	Completed	AML; Different types of leukemia; Erythroleukemia	Daunorubicin; Cytarabine; Clofarabine; Decitabine	≥60	3	
**NCT04022005**	Completed	Diffuse large B-cell lymphoma	Chidamide+Rituximab+Gemcitabine+Oxaliplatin	18-75	2	
**NCT06696378**	Recruiting	Female BC; Older adults; Exercise training program	Melatonin 6 mg (DS); Placebo (DS)	60-80	N/A	[605]
**Aging**						
**NCT04608448**	Completed	Aging; Epigenetics; Inflammatory mediators	Rapamycin topical ointment; Placebo	65-95	1	
**NCT04375657**	Recruiting	Epigenetic aging; Immunosenescence	TRIIM; Active control	40-80	2	
**NCT04946383**	Unknown	Aging	Dasatinib+Quercetin	≥40	2	[579]
**NCT05310123**	Unknown	Aging	AC-11 (DS)	≥55	2	
**NCT05110313**	Completed	Psoriasis; Aging; Epigenetic disorder, Skin inflammation	TILDRAKIZUMAB	≥35	4	
**NCT05964920**	Recruiting	Healthy aging; Age-related sarcopenia; Testosterone deficiency	Saline; Testosterone undecanoate; Saline+Resistance exercise training; Testosterone undecanoate+Resistance exercise training	55-70	2/3	
**NCT06411366**	Completed	Frailty; Aging	Follistatin plasmid	≥22	1	
**NCT05593939**	Completed	Aging	Exercise; Time restricted feeding; Nicotinamide riboside (DS)	≥65	2	
**NCT01754012**	Completed	Aging	Nutrient-rich diet+Cholecalciferol	65-79	N/A	[606]
**NCT06613542**	Active, not recruiting	Biological aging; Longevity; Physical activity; Metabolic health	Taurine; Placebo	55-75	N/A	
**NCT04110028**	Completed	Inflammation; Acute illness	Nicotinamide riboside; Placebo	≥18	1/2	
**NCT05294835**	Unknown	Depression; Post traumatic stress disorder	Ketamine	18-64	2	
**NCT05706389**	Unknown	Aging	Calcium Alpha-Ketoglutarate (Ca-AKG) (DS)	40-60	2	
**NCT06789900**	Recruiting	Postmenopausal women	Everolimus	45-60	N/A	

FdCyd: 5-Fluoro-2'-Deoxycytidine; AI: Aromatase inhibitor; AML: Acute myeloid leukemia; BC: Breast cancer; CML: Chronic myelomonocytic leukemia; CRC: Colorectal cancer; DLBCL: Diffuse large B cell lymphoma; DS: Dietary supplement; ER: Estrogen receptor; GBM: Glioblastoma multiforme; mCRPC: Metastatic castration-resistant prostate cancer; MDS: Myelodysplastic syndrome; NA: Not applicable; NSCLC: Non-small cell lung cancer; PDAC: Pancreatic ductal adenocarcinoma; PTCL: Peripheral T-cell lymphoma; SERD: Selective estrogen receptor degraders; THU: Tetrahydrouridine; TKI: Tyrosine kinase inhibitor; VPA: Valproic acid. Source: ClinicalTrials.gov, accessed on 2025/07/02.

### 3.7. CTs assessing cannabinoids

Nowadays, cannabinoids are indicated in cancer patients to ameliorate chemotherapy-induced nausea and vomiting, cancer-related pain, insomnia and mood disorders, anorexia and cachexia [544]. At present, there are 43 ongoing or completed CTs studying cannabinoids in cancer patients, 39 of them include older adults [545] both as palliative/supportive care or therapy, and 2 trials are now recruiting subjects to evaluate the role of cannabinoids in aging [546]. However, none of them are focused exclusively on older adults.

Of particular interest is cannabidiol, a non-psychoactive cannabinoid with anti-inflammatory properties, which improves healthspan parameters, including lessening age-related behavioral dysfunction, in models of both healthy and accelerated aging. These benefits likely occur through activation of antioxidative pathways and induction of autophagy [371]. A recent open-label, phase 1 dose-escalation study (NCT 04428203) in patients with recurrent PC following prostatectomy and/or salvage radiotherapy or primary definitive radiotherapy demonstrated the safety of cannabidiol at doses of 600 and 800 mg. Furthermore, at 12 weeks, 16 of 18 patients maintained stable biochemical markers [547]. Similarly, a randomized, placebo-controlled trial (NCT01812603/NCT01812616), involving 21 patients with recurrent glioblastoma multiforme, showed a longer survival when temozolomide was combined with nabiximols (Δ-9-tetrahydrocannabinol plus cannabidiol), compared with the combination of temozolomide with a placebo [548].

As highlighted in a recent review [549], most current preclinical and clinical studies focus on the interactions and potential risks of cannabinoids when combined with different anticancer therapies. Nevertheless, existing data also suggest that cannabinoids may increase the efficacy of chemotherapeutic agents while reducing their side effects. To date, most studies focus mainly on cytotoxicity, underscoring the need for additional research evaluating the effects of such combinations on the immune system, angiogenesis, invasion and metastasis.

CTs on the potential use of cannabinoids is of particular relevance in aged population, as it represents the fastest-growing group of medical marijuana users for the symptomatic management of various diseases and treatment-related AEs. To take advantage of the anticancer potential of cannabinoids in older adults, robust RCTs are required to evaluate the interactions between cannabinoids and the most common drugs taken by these polimedicated patients, including those for depression, cardiovascular disease, cholesterol, high blood pressure and blood thinning. These trials should also examine potential cognitive and psychological impacts, particularly in subjects with pre-existing conditions, as well as the effects on motor control.

In addition, given that the available data come mainly from studies performed in BC, glioblastoma and hematological malignancies, clinical research should be expanded to other cancer types with high incidence in older adults, such as lung, prostate and CRC. Given the interindividual variability in responses to cannabinoids, these trials should include large cohorts in order to generate high quality data on safety, efficacy and optimal cannabinoid dosing. Finally, considering the suggested impact of cannabinoids on aging process, long-term RCTs aimed at evaluating the use of these compounds to promote healthspan should be conducted.

## 4. Concluding remarks

Aging is considered one of the main risk factors for cancer development. Accordingly, the increase in cancer incidence is intimately associated with lifespan lengthening and with the consequent growth of the aging population. By 2050, the number of people aged 60 years and older is projected to reach 2.1 billion, with the number of subjects aged 80 years or older expected to reach 426 million. As a consequence, cancer incidence, especially among older adults, is expected to rise dramatically in the next years, representing one of the greatest challenges for public health systems.

In this context, elucidating the specific mechanisms that connect aging with carcinogenesis is of great importance. A better understanding of these mechanisms will not only help the development of more effective oncology therapies, but also of interventions aimed at delaying, reversing and stopping aging process itself.

There are many aging-associated changes in key physiological processes and systems that have been proposed to be on the root of cancer initiation and progression. These include somatic mutations, genome instability, DNA damage repair deficiency, aberrant microenvironment, senescence, telomere attrition, oxidative stress, defective adaptive and innate immunity, systemic inflammation, epigenetic changes, circadian clock disruption, dysfunctional endocannabinoid system, aberrant gut microbiome and metabolic dysregulation. The crosstalk between some of these systems and mechanisms (e.g., circadian clock-cell cycle, microbiome-circadian clock, ECS-immunosenescence) adds another layer of complexity to the picture.

In older adults, particular attention must be given to the selection of their treatments and to the preservation of mental and physical status during and after cancer therapy. The relevance of conducting CTs in older adults to issue evidence-based recommendations for clinical practice must be pointed out. Currently, there are several anti-aging therapies in older cancer patients that have been proven not only to improve patients’ fitness and QoL, but also to reduce chemo and radiotherapy adverse effects and/or to have oncological benefits. These interventions include lifestyle-related strategies, such as exercise and nutritional approaches (e.g., TRE), and pharmacological treatments, including CRMs, senolytics, senomorphics, epidrugs and cannabinoids, especially cannabidiol, given its anti-inflammatory properties. Probably, due to the reversible nature of the epigenetic modifications, one of the most promising anti-aging compounds to treat cancer and reverse drug resistance are the epidrugs, especially when given in combination with immune checkpoint inhibitors. Similarly, gut microbiome-based therapeutics for older subjects, aimed at reverting the unhealthy microbiome to a healthy one, represent a rapidly growing and promising area of research. Finally, given that many targets of anti-aging drugs display daily oscillations, it must be highlighted that dosage timing should be taken into account to optimize both efficacy and safety of anti-aging and oncological drugs.

Beyond anti-aging drugs discovery and clinical development, public health policies designed to promote prevention and healthy aging must also be prioritized.
